# The expressed mutational landscape of microsatellite stable colorectal cancers

**DOI:** 10.1186/s13073-021-00955-2

**Published:** 2021-09-01

**Authors:** Anita Sveen, Bjarne Johannessen, Ina A. Eilertsen, Bård I. Røsok, Marie Gulla, Peter W. Eide, Jarle Bruun, Kushtrim Kryeziu, Leonardo A. Meza-Zepeda, Ola Myklebost, Bjørn A. Bjørnbeth, Rolf I. Skotheim, Arild Nesbakken, Ragnhild A. Lothe

**Affiliations:** 1 Department of Molecular Oncology, Institute for Cancer Research, Oslo University Hospital, P.O. Box 4953 Nydalen, NO-0424 Oslo, Norway; 2 K.G. Jebsen Colorectal Cancer Research Centre, Division for Cancer Medicine, Oslo University Hospital, P.O. Box 4953 Nydalen, NO-0424 Oslo, Norway; 3 Institute of Clinical Medicine, Faculty of Medicine, University of Oslo, P.O. Box 1171 Blindern, NO-0318 Oslo, Norway; 4 Department of Gastrointestinal Surgery, Oslo University Hospital, P.O. Box 4950, NO-0424 Oslo, Norway; 5 Department of Tumor Biology, Institute for Cancer Research, Oslo University Hospital, P.O. Box 4953 Nydalen, NO-0424 Oslo, Norway; 6 Genomics Core Facility, Department of Core Facilities, Institute for Cancer Research, Oslo University Hospital, P.O. Box 4953 Nydalen, NO-0424 Oslo, Norway; 7 Department of Clinical Science, University of Bergen, P.O. Box 7804, NO-5020 Bergen, Norway; 8 Department of Informatics, Faculty of Mathematics and Natural Sciences, University of Oslo, P.O. Box 1032 Blindern, NO-0315 Oslo, Norway

**Keywords:** Colorectal cancer, Exome sequencing, RNA sequencing, Allele-specific mutation expression, Mutant allele fraction, Pharmacogenomics, Drug screening, Patient-derived organoids

## Abstract

**Background:**

Colorectal cancer is the 2nd leading cause of cancer-related deaths with few patients benefiting from biomarker-guided therapy. Mutation expression is essential for accurate interpretation of mutations as biomarkers, but surprisingly, little has been done to analyze somatic cancer mutations on the expression level. We report a large-scale analysis of allele-specific mutation expression.

**Methods:**

Whole-exome and total RNA sequencing was performed on 137 samples from 121 microsatellite stable colorectal cancers, including multiregional samples of primary and metastatic tumors from 4 patients. Data were integrated with allele-specific resolution. Results were validated in an independent set of 241 colon cancers. Therapeutic associations were explored by pharmacogenomic profiling of 15 cell lines or patient-derived organoids.

**Results:**

The median proportion of expressed mutations per tumor was 34%. Cancer-critical mutations had the highest expression frequency (gene-wise mean of 58%), independent of frequent allelic imbalance. Systematic deviation from the general pattern of expression levels according to allelic frequencies was detected, including preferential expression of mutated alleles dependent on the mutation type and target gene. Translational relevance was suggested by correlations of *KRAS*/*NRAS* or *TP53* mutation expression levels with downstream oncogenic signatures (*p* < 0.03), overall survival among patients with stage II and III cancer (*KRAS*/*NRAS*: hazard ratio 6.1, *p* = 0.0070), and targeted drug sensitivity. The latter was demonstrated for EGFR and MDM2 inhibition in pre-clinical models.

**Conclusions:**

Only a subset of mutations in microsatellite stable colorectal cancers were expressed, and the “expressed mutation dose” may provide an opportunity for more fine-tuned biomarker interpretations.

**Supplementary Information:**

The online version contains supplementary material available at 10.1186/s13073-021-00955-2.

## Background

Mutation profiling is routinely used in several cancer types to guide the selection of targeted therapies for patients. However, precision oncology guided by genomics has been less useful than anticipated and was estimated to benefit less than 7% of cancer patients in 2018 [1]. Colorectal cancer (CRC) is the second most common cause of cancer deaths worldwide [2], and the patients have few molecularly guided treatment options [3, 4]. Beyond the microsatellite instability phenotype as a cause of a high tumor mutational burden (TMB) and a marker of response to immune checkpoint inhibition [5, 6], diagnostic mutation profiling is currently limited to *KRAS*/*NRAS* (*RAS*) exons 2–4, which are mutations associated with resistance to monoclonal anti-EGFR antibodies, and to *BRAF*^V600E^ as a target for combination therapies [7]. The efficacy of the anticancer agents varies even in biomarker-selected populations, and more detailed molecular pre-screening is needed. Examination of mutation levels may increase the accuracy of response predictions. This has been illustrated by an inverse correlation between the allelic fractions of *RAS*/*BRAF*^V600E^/*PIK3CA* mutations and response to anti-EGFR therapy [8].

Microsatellite stable (MSS) CRCs have a median mutation rate of approximately 3 mutations per megabase [9]. However, the number of mutated driver genes that confer a selective growth advantage is limited [10], and the vast majority of somatic mutations are likely insignificant passenger events. The expression of the mutated allele is a determinant of its functional consequences, and integrated genomic and transcriptomic analyses can aid in the prioritization of cancer-critical mutations [11, 12]. The genome of CRCs has more frequent allele-specific expression regulation than matched normal colonic tissue [13], likely related to DNA copy number aberrations and allelic imbalance [14]. In light of this, surprisingly little has been done to analyze somatic mutations on the expression level.

The few studies that have investigated cancer mutation expression on a genome-wide scale have reported that the proportion of mutations that are expressed ranges from 27% in multiple myelomas [15] to 40% across non-small cell lung cancers [16] and 59% in breast cancers [12]. The large variation is likely associated with sample selection and the small number of cancers analyzed in each study (≤ 14), as well as by technical factors such as sequencing depth [15] and bioinformatic data processing. Current data, also including data from three cell lines of different cancer types [17], indicate that allele-specific expression levels at mutated loci correspond with the mutant allele fraction (MAF) on the DNA level. However, the potential regulation of mutations on the expression level has not been systematically investigated in large cancer series.

In this study, we combined whole-exome and RNA sequencing to map the landscape of mutation expression according to the allelic fraction in a total of 362 MSS CRCs. We also performed proof-of-concept analyses of potential therapeutic associations in pre-clinical models.

## Methods

### Patient material

Patient samples were from an ongoing prospective observational study of patients treated by major resection for primary CRC at Oslo University Hospital, Norway, after December 2005. The study involves the collection of fresh frozen samples of the tumor and adjacent normal colonic mucosa from surgical specimens, and the series is population-representative for the south-east of Norway. Patients were treated according to the national guidelines, including pre-surgical radiotherapy or chemoradiation for locally advanced rectal cancers, and adjuvant 5-fluorouracil-based combination chemotherapies with leucovorin and oxaliplatin according to cancer stage, patient age, and tolerability. Patients in the current study (*n* = 121) were selected to include only MSS tumors diagnosed predominantly as stage II or III cancers (89%) between December 2005 and August 2010, with no residual tumor in the colon/rectum after elective surgery (except one patient with microscopic resection margin less than 1 mm), no in-hospital mortality, and no treatment prior to surgery (except four patients with pre-operative radiotherapy). Fifteen of the patients (initially diagnosed between September 2010 and March 2016) were also included in a separate ongoing prospective observational study of patients admitted for hepatic resection of colorectal liver metastases at Oslo University Hospital after October 2013 [18]. Multiple tissue samples were included from four of these patients for analysis of tumor heterogeneity, including 2–3 spatially separated samples from the primary tumor of three patients (in a total of 8 multiregional primary tumor samples) and 11 samples from 5 liver metastases from four patients. All primary tumor samples (*n* = 126 samples from 121 tumors and patients) were included for all analyses unless otherwise stated. This is referred to as the in-house series, and clinicopathological characteristics are summarized in Table 1. Liver metastasis samples were included only for separate analysis of tumor heterogeneity. DNA/RNA extraction and determination of microsatellite instability status have previously been performed [18, 19]. Extraction was either based on a magnetic bead approach using the Maxwell 16 DNA Purification Kit (DNA) or the Qiagen AllPrep DNA/RNA/miRNA Universal kit (DNA/RNA), both according to the manufacturers’ instructions (Promega, Madison, WI, USA, and Qiagen, GmBH, Hilden, Germany, respectively).

**Table 1:** Clinicopathological characteristics and expressed TMB of the in-house series of primary MSS CRCs

	Patients with MSS CRC (*n* = 121)	TMB^a^		Expressed TMB^a^	
		Mean [95% CI]	*p*	Mean [95% CI]	*p*
All tumors	121 (100%)	161 [147–174]	–	38 [35–42]	–
Gender					
Male	63 (52.1%)	156 [139–173]	0.44	37 [33–42]	0.49
Female	58 (47.9%)	166 [145–188]		40 [34–46]	
Age, median [10–90th]	72.5 [54.7–85.4]				
Above median age		158 [141–175]	0.71	38 [34–43]	0.99
Below median age		163 [142–185]		39 [34–44]	
Tumor localization^b^					
Right	51 (42.1%)	177 [155–198]	0.088^c^	44 [38–50]	0.037^c^
Left	42 (34.7%)	155 [134–175]		36 [31–40]	
Rectum	27 (22.3%)	142 [110–175]		33 [26–40]	
Synchronous	1 (0.8%)	–		–	
Cancer stage					
I	1 (0.8%)	–	–	–	–
II	59 (48.8%)	166 [148–183]	Reference	39 [34–45]	Reference
III	49 (40.5%)	170 [145–195]	0.50^d^	39 [34–45]	0.89^d^
IV	12 (9.9%)	107 [87–127]	0.0003^d^	32 [23–40]	0.099^d^
Treatment prior to tumor sampling					
Yes^e^	4 (3.3%)	61 [− 9–131]	0.014	12 [–7–31]	0.016
No	117 (96.7%)	164 [151–178]		39 [36–43]	
Adjuvant chemotherapy (non-available: *n* = 1)					
Yes	36 (30%)	158 [128–189]	0.2	37 [30–43]	0.2
No	84 (70%)	162 [147–177]		39 [35–43]	

^a^Non-synonymous SNVs, frameshift indels, splice site mutations (for 3 of the tumors: mean of multiregional samples)

^b^Tumors in the transverse colon (*n* = 8) were considered right-sided

^c^Right versus left and rectum, based on the Mann-Whitney *U*-test, excluding four rectal tumors treated with pre-operative radiotherapy (including the synchronous)

^d^Mann-Whitney *U*-test with stage II as a reference category

^e^Pre-operative radiotherapy for locally advanced rectal cancer

Data from MSS colon cancers in The Cancer Genome Atlas (TCGA [9]) were used for independent validation analyses, and the validation series included 241 primary tumors of stage I–IV cancers (excluding six tumors with *POLE*-associated hypermutation and one tumor in which 59% of mutations were insertions or deletions [indels], all with a TMB above 1600). Clinicopathological characteristics and MSS status have previously been obtained using the Broad Institute Firehose tool (https://gdac.broadinstitute.org/ in March 2017) and are summarized in Additional file 1: Table S1. Additional molecular data files were obtained as described below and matched based on the patient barcode.

### Whole-exome sequencing and mutation calling

Whole-exome sequencing was performed on patient-matched tumor and normal colonic mucosa samples to a mean depth of 311 (10–90th percentile among the primary tumor samples 168–472) and 171 (10–90th percentile 94–182) times coverage, respectively. Exome libraries were generated from 1 μg of genomic DNA using the Agilent SureSelect Human All Exon v5 or v6+COSMIC kits (Agilent, Santa Clara, CA, USA), and sequencing was performed with the Illumina HiSeq 2500/4000 system (Illumina, San Diego, CA, USA) in 2 × 100 base-pair paired-end mode at the Oslo University Hospital Genomics Core Facility (The Norwegian Radium Hospital, Oslo, Norway). Processing of raw sequencing reads was done according to our previously described bioinformatics pipeline [20], including sequence alignment to the GRCh37 human reference genome using BWA, file format manipulations, and filtering of sequencing reads using Samtools, Picard, and GATK, as well as mutation calling with MuTect (single nucleotide variants (SNVs)) and Strelka (indels), mutation annotation by Annovar, and conversion of variants to the GRCh38 genome reference using the LiftoverVcf-function in Picard. Candidate somatic mutations were filtered to include only loci with MAF ≥ 5%, and at least 15 times and 10 times coverage in the tumor and matched normal sample, respectively. A single variant read in the normal sample was accepted. Mutations in *KRAS* and *BRAF*^V600E^ were verified by Sanger sequencing, as previously described [21].

Mutations were categorized as amino acid changing (non-synonymous exonic SNVs [missense, nonsense, stoploss], frameshift indels, and splice site mutations [SNVs, indels]) or non-amino acid changing (exonic synonymous SNVs and inframe indels). The total number of detected mutations across the 126 primary tumor samples was 28,474, and the number of amino acid changing mutations was 19,989 (Additional file 1: Table S2).

Amino acid changing mutations were classified as cancer-critical if found in genes included in the Cancer Gene Census [22] (CGC; tiers 1 and 2; downloaded from https://cancer.sanger.ac.uk/census in March 2019). Further classification as oncogenes and/or tumor suppressor genes was adopted from the CGC if based on relevant mutation types (missense, nonsense, frameshift, or splice site mutations). Genes included in the CGC based on other mutation types (translocations, amplification, large deletion, others) were classified as “CGC other.” Mutated genes were categorized as “FLAGS” if included in a list of genes (*n* = 100) for which frequent mutations detected in exome sequencing studies have been associated with common features such as a long protein-coding sequence and a large number of paralogs [23].

### RNA sequencing and gene expression estimation

RNA sequencing of all in-house tumor samples, a subset of the normal colonic mucosa samples (*n* = 12), and pre-clinical CRC models (described below) was performed in a 2 × 101 base-pair paired-end mode on the Illumina HiSeq 2500/4000 platform. Sample preparation, including ribosomal RNA depletion using the Ribo-Zero Gold rRNA removal kit and sequence library generation with the TruSeq Stranded Total RNA Library Prep Gold kit (Illumina), was done at the Oslo University Hospital Genomics Core Facility. Bioinformatic processing of raw sequencing reads was done as previously described [24], including adapter trimming with Trimmomatic version 0.38, alignment to the human reference genome GRCh38 using STAR, read sorting by SAMtools, and quantification of reads mapping to protein-coding genes using the HTSeq-count tool (version 0.10.0). The median number of uniquely mapped trimmed RNA sequencing read pairs across the 126 primary tumor samples was 30.2 × 10^6^ (10–90th percentile 24.7 × 10^6^–50.2 × 10^6^).

Sample-wise normalization of gene expression levels was done by estimation of the fragments per kilobase of transcripts per million mapped reads (FPKM). The distinction between active genes and background expression was defined by zFPKM transformation of the expression matrix using the R package zFPKM [25]. Genes with zFPKM ≤ − 3 were defined as non-expressed. The median proportion of non-expressed genes across the 126 primary tumor samples was 28% (10–90th percentile 26–31%). Cross-sample normalization was performed by voom-transformation [26] of the trimmed mean of *M* values [27] using the R package edgeR [28].

### Allele-specific expression at mutated loci

Allele-specific expression analysis at each mutated locus was performed by sample-wise integration of whole-exome and RNA sequencing data using the ASEReadCounter function [29] included in the GATK toolkit (version 3.8; https://software.broadinstitute.org/gatk/). VCF files from exome sequencing and BAM files from RNA sequencing were used as an input. ASEReadCounter was run with the additional parameters min-mapping-quality = 10, min-base-quality = 2, and U = ALLOW_N_CIGAR_READS. Outputs were sample-wise RNA read counts of both the mutated and wild-type alleles at SNV loci specified in the VCF files, after filtering based on the data quality parameters. Allele-specific RNA read counts at indel loci were calculated using the SAMtools mpileup command. The mutated loci were additionally filtered by coverage in the RNA sequencing data based on the zFPKM values of the mutated genes and the total read count at the mutated position from ASEReadCounter, as specified in Additional file 1: Table S3. Of the 28,474 total mutations and 19,989 amino acid changing mutations detected in the 126 primary tumor samples, 19,981 (70.2%) and 14,228 (71.2%) were included for allele-specific expression analyses, respectively. Mutations were categorized as expressed if the MAF in the RNA sequencing data was ≥ 5% (mutated allele expressed) and non-expressed if either the RNA MAF was < 5% (mutated allele not expressed) or the zFPKM of the mutated gene was ≤ − 3 (mutated gene not expressed).

For gene-wise summarization of mutation frequencies, each mutated gene was counted once per sample (including 19,069 of the total 19,989 amino acid changing mutations in the primary tumor samples). For genes with multiple mutations per sample, the expressed mutations were included.

For comparison of allele-specific mutation expression levels across samples, the RNA sequencing read counts of the mutated alleles were normalized by the sequencing depth of the sample: [read count_mutated allele_/number of uniquely mapped reads] × 10^6^.

### Regulation of mutations at the allele-specific expression level

The MAF at the RNA level (proportion of RNA sequencing reads representing the mutated allele) was calculated to analyze the potential preferential or reduced expression of the mutated versus corresponding wild-type allele. The RNA MAF was compared with the DNA MAF from the exome sequencing data to adjust for the allelic fraction on the DNA level. A possible bias in these comparisons was related to the sequencing of DNA and RNA that did not originate from the same extraction of 115 of the 126 primary tumor samples. Neighboring tissue samples were used to minimize the potential influence from intra-tumor heterogeneity. Furthermore, data correction was performed assuming that the majority of expressed mutations have no allele-specific expression regulation, that is, the RNA MAF is proportional to the DNA MAF: all RNA MAFs were divided by a sample-wise adjustment factor calculated as the median [RNA MAF/DNA MAF] of all expressed mutations in the sample (RNA MAF_adjusted_). The appropriateness of this adjustment was evaluated by comparing RNA MAFs from two repeated RNA extractions of 3 tumors (MAF adjustments needed for one of the samples per tumor), confirming high tumor-wise Pearson’s correlations (0.73–0.87, *p* < 3 × 10^−5^). For additional quality control, the results were compared with samples for which combined DNA and RNA extraction was performed (a total of 11 samples from 6 tumors, including all 8 multiregional samples from 3 tumors). In total, 17 (13.5%) of the primary tumor samples were excluded from the analyses of allele-specific mutation expression levels (including two of the four tumors treated with pre-operative radiotherapy), either due to few expressed mutations (< 12) or a high adjustment factor (median [RNA MAF/DNA MAF] > 1.75). Among the remaining tumors (*n* = 109), the median adjustment factor was 1.22 (10–90th percentile 0.99–1.54). For reference, the median adjustment factor among samples with DNA and RNA from the same extraction procedure was 1.24 (10–90th percentile 1.04–1.54).

### DNA copy number estimation

Allele-specific DNA copy numbers and tumor purity were estimated from the exome sequencing data using paired tumor-normal BAM files as input for the R package FACETS with default settings [30]. The mutated loci with an equal number of copies of the mutated and wild-type allele were considered balanced. Other loci were considered to have an allelic imbalance. Notably, the term allelic imbalance was used only with reference to the DNA-level data. Allele-specific data on the RNA level were referred to as allelic expression.

### Independent validation data set

A list of mutations from whole-exome sequencing of the validation series of 241 primary MSS colon tumors from TCGA was downloaded using the R package TCGABioLinks [31] and filtered based on the same criteria for MAF and coverage as in the in-house data set. Additional filtering was performed to exclude mutation categories not annotated in the in-house data (downstream/upstream gene variants, 3′/5′ UTR variants, intergenic variants, intronic variants, non-coding transcript/miRNA variants), retaining a total of 31,448 mutations and 23,493 amino acid changing mutations. Paired tumor-normal BAM files were also downloaded and used as input for allele-specific copy number estimation by FACETS. Allele-specific expression analyses were carried out as for the in-house data set, and VCF files from exome sequencing (mutation calling with Mutect2) and BAM files from RNA sequencing were downloaded from the NCI’s Genomics Data Commons [32] Data Portal (https://portal.gdc.cancer.gov; December 2019). FPKM values estimated from RNA read counts quantified by HTSeq-count were also downloaded. Based on the same criteria as for the in-house data set, 20,351 (64.7%) mutations in total and 15,164 (64.5%) amino acid changing mutations were included for allele-specific expression analyses (Additional file 1: Table S3). For cross-sample comparisons, RNA sequencing read counts of mutated alleles were normalized by the sequencing depth, and the number of uniquely mapped reads per sample was calculated from the BAM files using SAMtools with the following grep command: samtools view <SAMPLE>.bam | grep –cw ‘NH:i:1’.f

### Drug sensitivity and mutation expression in pre-clinical models

Fifteen pre-clinical models with resistance mutations, including CRC cell lines (*n* = 7; CACO2, COLO205, HCC2998, IS1, NCIH508, SW1116, and SW948) and patient-derived organoids (PDOs) of CRC liver metastases (*n* = 8), were analyzed for drug sensitivity in relation to mutation expression. Specifically, the associations between the gene-drug pairs *RAS*/*BRAF*^V600E^ mutations and EGFR or MEK inhibition, as well as *TP53* mutations and MDM2 inhibition, were investigated. The cell lines were selected from an in-house collection of 29 cell lines, for which targeted next-generation DNA sequencing (including of *RAS*, *BRAF*, and *TP53* [33]) and high-throughput drug sensitivity screening [19, 34] have previously been published. PDOs were selected from a collection of 39 PDOs established from distinct liver lesions of 22 patients treated by hepatic resection for metastatic CRC at Oslo University Hospital between 2017 and 2019. In short, organoids were cultured in Matrigel (Corning) overlaid with ENAS media [35] (supplemented with the ROCK inhibitor Y-27632, Selleck Chemical, Houston, TX, USA, in the initial growth phase), screened for sensitivity to 40 anticancer agents (450–600 strained organoids in 3% Matrigel/ENAS media supplemented with the ROCK inhibitor were seeded to each well of the drug screen plates), and analyzed by Sanger sequencing of *RAS*, *BRAF*^V600E^ and *TP53*, all as previously described [36]. The MDM2-TP53 inhibitor idasanutlin (MedChemExpress, Monmouth Junction, NJ, USA), three EGFR inhibitors (afatinib: Selleck Chemicals; erlotinib: MedChemExpress; and lapatinib: LC Laboratories, Woburn, MA, USA) and two MEK inhibitors (binimetinib and trametinib, ChemieTek, Indianapolis, IN, USA) were included in the screens at five and nine different concentrations over a 10,000-fold concentration range each (typically 1–10,000 nmol/L) in the cell lines and PDOs, respectively. Drug sensitivity scores (DSS [37]) were calculated based on cell viability (CellTiter-Glo assay; Promega, Fitchburg, WI, USA) after 72 and 96 h of drug exposure in cell lines and PDOs, respectively, and relative to negative (0.1% DMSO) and positive controls (100 μmol/L benzethonium chloride). Additional details of the growth protocols and drug sensitivity screens have previously been described [34, 36]. Among the EGFR inhibitors, afatinib and erlotinib had strongly correlated sensitivity levels within the selected cell lines (Spearman’s *ρ* = 0.96, *p* = 0.003) and PDOs (Spearman’s *ρ* = 0.9, *p* = 0.005). The two MEK inhibitors were also correlated (cell lines: Spearman’s *ρ* = 0.68, *p* = 0.11; PDOs: Spearman’s *ρ* = 0.90, *p* = 0.005). Erlotinib and trametinib were chosen for data presentation based on high cross-sample variance among PDOs.

The 15 pre-clinical models were selected for RNA sequencing from the larger sets of samples based on MSS status, *RAS*/*BRAF*^V600E^, and/or *TP53* mutation status, as well as for representing a large range in DSS of the relevant drugs. RNA sequencing was performed as described above. Targeted analyses of allele-specific read counts at the relevant mutated loci were performed using the SAMtools mpileup command. For cross-sample comparisons, RNA read counts of the mutated alleles were normalized by the RNA sequencing depth of the sample: [read count_mutated allele_/number of reads mapped to protein-coding genes] × 10^6^.

### Statistical analyses

All statistical analyses were performed in R v.3.6.1. All *p*-values were two-sided. Welch’s *t*-test and paired samples *t*-test were performed with the function t.test, Mann-Whitney *U*-test with the function wilcox.test, correlation analyses with the function cor.test (Pearson’s *r* or Spearman’s *ρ* correlation coefficients as appropriate), Fisher’s exact test with fisher.test, and 95% confidence intervals (CIs) for the mean and median were calculated using the R packages Rmisc and DescTools, respectively. Cox proportional hazards analyses were performed with the survival R package, and *p*-values were calculated using the Wald test. Kaplan-Meier plots were made with the survminer package, and *p*-values were calculated using the log-rank test. Survival analyses included patients with stage II or III CRC in the in-house series, and the endpoint was 5-year overall survival. Single-sample gene set enrichment analyses of the hallmark gene set collection (*n* = 50 gene sets retrieved from the Molecular Signatures Database [38]), as well as expression signatures of *KRAS* mutations [39] and *TP53* mutations (in-house signature based on 5 genes with high expression in wild-type compared to *TP53* mutated primary CRCs: *MDM2*, *SPATA18*, *FAS*, *DDB2*, *HSPA4L* [36]), were done using the “ssgsea” method in the R package GSVA [40]. Input was log2-transformed FPKM values after the addition of a constant of 1 to avoid infinite values for genes with FPKM = 0. Enrichment analyses of lists of genes with expressed mutations were performed using the Enrichr web server [41], querying the Reactome pathway database. Binary dimensionality reduction of a binary expressed mutation matrix of samples and genes (expressed mutation 1 or 0) was performed by logistic principal component analysis (PCA) using the R package logisticPCA [42], with parameter *k* = 2 and the optimal m determined by cross validation. Density plots of distributions within mutation, gene, or sample groups were made using the R package ggplot2 for illustration purposes only and were not used for statistical analyses. Matrices of scatter plots were drawn with the pairs.panels function in the R package psych.

## Results

### Heterogeneity of expressed tumor mutational burden of MSS CRCs

**Fig. 1:**
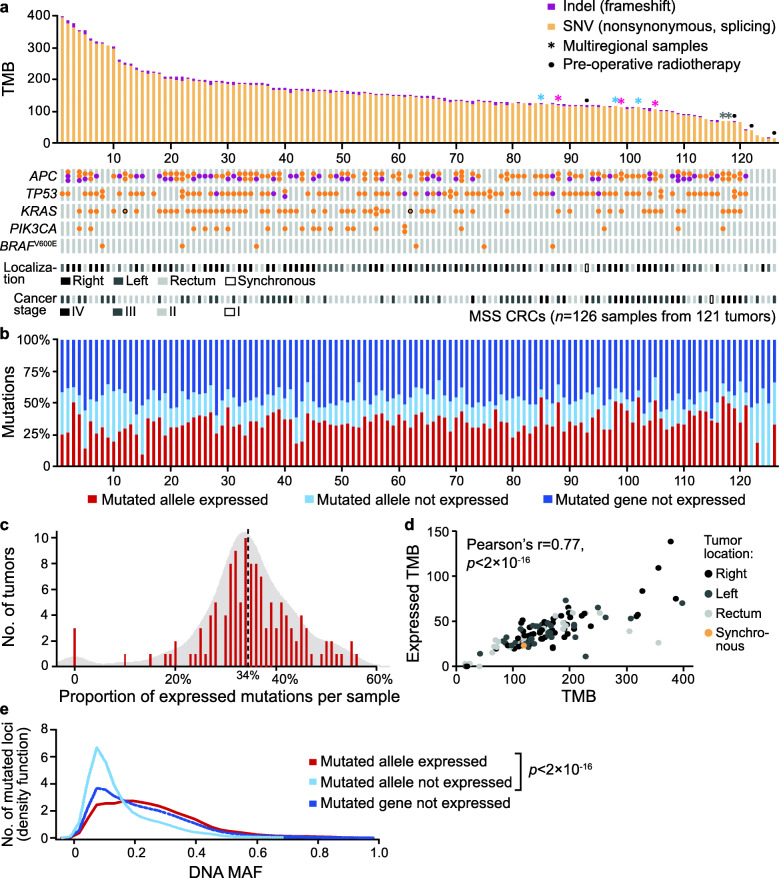
Heterogeneity in the expressed tumor mutational burden of primary MSS CRCs. **a** Range of TMBs of 121 primary MSS CRCs, including 8 multiregional samples from 3 tumors (indicated by asterisks colored according to tumor-of-origin). The bottom panel shows sample-wise mutations in five selected genes (two *KRAS* mutations detected by Sanger sequencing only are indicated with black outlines), tumor localization, and cancer stage. **b** Sample-wise proportion of the mutated loci with expression of the mutated allele. Non-expressed mutations were found either at the loci with exclusive expression of the wild-type allele or in non-expressed genes. The tumors are plotted in the same order as in **a**. **c** Frequency plot showing the median (dashed line) sample-wise proportion of expressed mutations. **d** Scatter plot showing correspondence between the expressed TMB and TMB, colored according to tumor location. **e** Density plot of DNA MAFs for mutations grouped according to expression category. In **b**, **c**, and **e**, only the mutated loci covered in the RNA sequencing data were included for calculations, while **d** shows the non-filtered TMB (corresponding with **a**)

A total of 126 primary tumor samples from an in-house series of 121 MSS CRCs, including 8 multiregional samples from 3 tumors, were initially analyzed by whole-exome and total RNA sequencing (Table 1). The median TMB (number of non-synonymous and frameshift mutations per tumor) was 151 (95% CI 133–164; Fig. 1a and Additional file 1: Table S2), corresponding to a median of 3.0 mutations per sequenced megabase (95% CI 2.7–3.3; Additional file 2: Figure S1). The TMB was not associated with the exome sequencing depth (Pearson’s *r* = 0.05; Additional file 2: Figure S1), but four rectal tumors treated with pre-operative radiotherapy showed a significantly lower TMB, likely related to fibrotic tissue and poor data quality (Table 1). Right-sided tumors had a higher TMB than left-sided or rectal tumors, also when excluding pre-treated tumors, although the difference was small and non-significant. The TMB was not associated with patient gender or age but was lower in tumors from stage IV compared to stage II or III cancers (Table 1). Cox proportional hazards analyses showed no associations with 5-year overall survival among the 108 patients with stage II or III CRC, neither in univariate analysis nor in a multivariable model including the parameters listed in Table 1 (*p* ≥ 0.1 for the TMB as a continuous variable). The list of the most frequently mutated genes corresponded well with the known mutation profiles of MSS CRCs (Additional file 2: Figure S2).

The median proportion of non-synonymous and frameshift mutations that was expressed per tumor was only 34% (95% CI 33–36; Fig. 1b, c). This proportion was weakly associated with the tumor purity of the samples (estimated based on DNA copy numbers) and RNA sequencing depth (Additional file 2: Figure S1), but the association with the TMB was stronger, with a Pearson’s correlation between the TMB and expressed TMB of 0.77 (*p* < 2 × 10^−16^, Fig. 1d; TMB after filtering based on RNA sequence coverage: Pearson’s *r* = 0.85). The failure to detect expressed mutations in 3 of the tumors was likely accounted for by the low TMB (≤ 41 mutations) and low DNA MAFs (Additional file 2: Figure S1). The expressed TMB was higher in right-sided compared to left-sided or rectal MSS CRCs, but not significantly lower in stage IV compared to earlier stage cancers (Table 1). Notably, there was no significant difference in the proportion of mutations that were expressed among tumors stratified according to localization or cancer stage. Single-sample gene set enrichment analysis showed that the expressed TMB was most strongly correlated to proliferative signatures (hallmark gene set collection; Additional file 2: Figure S3a), but the expressed TMB was not associated with patient survival (*p* > 0.3). Validation analyses in MSS colon cancers from TCGA (primary tumor from *n* = 241 stage I–IV cancers) showed similar results, with a median proportion of expressed mutations of 39% (95% CI 38–41), a Pearson’s correlation between the TMB and expressed TMB of 0.68 (*p* < 2 × 10^−16^), and a significantly higher expressed TMB in right-sided compared to left-sided colon cancers (mean of 26 and 22, respectively, *p* = 0.006), but no difference in the TMB or expressed TMB according to cancer stage (Additional file 1: Table S1).

The majority of non-expressed mutations were found in inactive, non-expressed genes (median of 47% [95% CI 44–48] of all mutations per sample in the in-house series). Furthermore, several mutated loci in expressed genes had expression exclusively of the wild-type allele (median sample-wise proportion of 18%, 95% CI 17–19; Fig. 1b), and the mutations at these loci had lower DNA MAFs (mean 15%) than loci with expressed mutations (mean 25%, *p* < 2 × 10^−16^; Fig. 1e). The results were similar when analyzing only mutations at balanced DNA copy number loci (equal number of copies of the mutated and wild-type allele; corresponding mean difference in MAF of − 8 percentage points between loci with wild-type only versus mutant allele expression, *p* < 2 × 10^−16^), and consistent in sample-wise analysis (mean paired difference − 9, *p* < 2 × 10^−16^; paired samples t-test), suggesting independence of potential confounders such as allelic imbalance and tumor purity of the samples, respectively. A similar difference in DNA MAFs at loci with wild-type only compared to mutated allele expression was also found in the TCGA series, including at copy number balanced loci (Additional file 2: Figure S4a).

### Mutations in cancer-critical genes are more frequently expressed

**Fig. 2:**
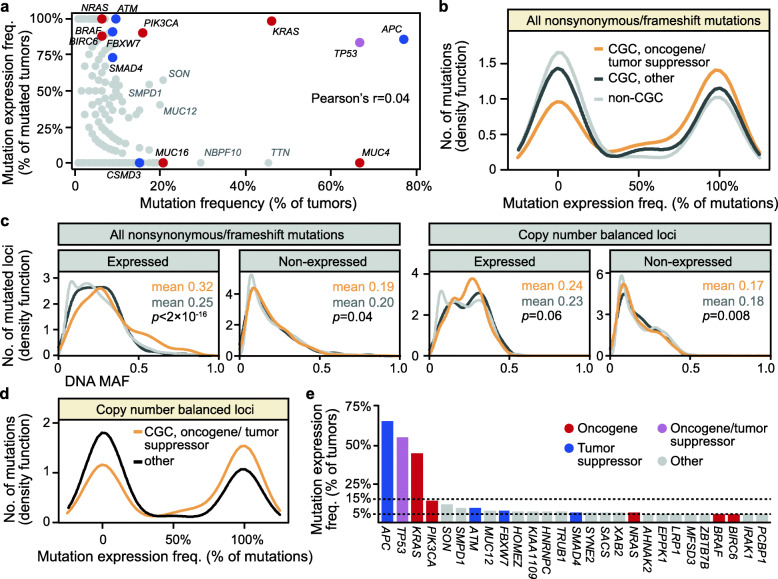
Mutations in cancer-critical genes are more frequently expressed. **a** The gene-wise proportion of mutated loci with mutant allele expression (calculated relative to all tumors in the in-house series with non-synonymous SNVs, frameshift indels, or splice site mutations in each gene) is plotted on the vertical axis, and the mutation frequency on the horizontal axis. Selected genes are indicated with names and colored according to the labels defined in **e**. **b** Density plot of the proportion of mutations with expression of the mutated allele, grouped according to target gene category (oncogenes/tumor suppressor genes were defined by the Cancer Gene Census (CGC)). **c** Density plot of DNA MAFs grouped according to target gene category and plotted separately for expressed and non-expressed mutations, as well as for mutations at copy number balanced loci only. **d** Density plot of the proportion of mutations at copy number balanced loci with the expression of the mutated allele, grouped according to target gene category. **e** Frequency plot of the 27 genes with expressed mutations in more than 5% of the tumors, plotted in decreasing order

The frequency of mutation expression was also dependent on the target gene, and summarized per gene, the proportion of mutated tumors with expression of the mutated allele ranged from 0 to 100% (Fig. 2a). This was independent of the mutation frequency of the gene, but mutations in oncogenes or tumor suppressor genes (defined by the CGC [22]) were more frequently expressed (mutations in each gene were expressed in a mean of 58% of the mutated tumors, 95% CI 52–64) than mutations in other genes (mean 39%, 95% CI 38–41; *p* = 6 × 10^−9^; Fig. 2b). Again, this was associated with higher DNA MAFs specifically of expressed (not of non-expressed) mutations in oncogenes/tumor suppressor genes than in other genes, both in the in-house series (Fig. 2c) and the TCGA data (Additional file 2: Figure S4b). However, mutated loci in cancer-critical genes were also somewhat more frequently targeted by allelic imbalance than other mutations (odds ratio [OR] 1.2, *p* = 0.004 by Fisher’s exact test), and there was no clear difference in DNA MAFs according to target gene category for mutations (expressed or non-expressed) at copy number balanced loci (Fig. 2c). Nonetheless, the mutation expression frequency (proportion of mutated tumors with the mutation expressed) was higher in oncogenes/tumor suppressor genes also at balanced loci (mean mutation expression frequency of 57% versus 38% in other genes, *p* = 2 × 10^−6^; Fig. 2d; validation in TCGA data; Additional file 2: Figure S4c), suggesting that allelic imbalance is not a sole determinant of frequent expression of cancer critical mutations.

A heatmap of the expressed mutation matrix (expressed mutation: yes/no) showed no distinct patterns among samples or genes, indicating that genes contributed to the expressed TMB in accordance with their mutation expression frequency (Additional File 2: Figure S5). Three genes had expressed mutations in a particularly large proportion of tumors including the well-known CRC-critical genes *APC* (66%), *TP53* (56%), and *KRAS* (45%; Fig. 2e). The remaining genes (*n* = 3,017) contributed to a “long tail” of potentially functional cancer mutations, with mutation expression in less than 15% of tumors each (99% of genes in less than 5% of tumors, and 71% of genes were non-recurrent among the 126 tumor samples; Additional file 1: Table S4). *APC*, *TP53*, and *KRAS* were outlier genes also in logistic PCA of the binary expressed mutation matrix, consistent with a strong correlation between PC1 and the expressed mutation frequency per gene (Additional file 2: Figure S6). The distinction of genes along PC2 appeared to be driven by the co-occurrence of their expressed mutations with mutation expression in either *KRAS* or *TP53*. Exploratory analysis of genes not designated as oncogenes and/or tumor suppressors, but with frequently expressed mutations, indicated enrichment with gene sets related to mRNA splicing (*HNRNPC* and *PCBP1*) and deactivation of β-catenin (*TCF7L2* [implicated in CRC as fusion gene target], *SOX9*, and *SOX4*; Additional file 2: Figure S3b).

Frequently mutated but non-expressed genes were overrepresented among genes with features of non-pathogenic mutation accumulation, such as a long coding sequence (OR 24.2, 95% CI 11.5–48.0; Additional file 2: Figure S7) [23]. The majority of these target genes also had low expression levels in wild-type tumors and/or normal colonic mucosa samples, indicating that the mutations were not selectively silenced. Similarly, the mutated loci with wild-type only allelic expression were not associated with large variation in target gene expression between mutated and wild-type tumors (with the notable exception of *COL12A1*; Additional file 2: Figure S8). However, mutations that were expressed in a subset of mutated tumors, and silenced in others, showed a varying pattern of target gene expression according to mutation status, suggesting allele-specific expression regulation (for example in *ATRX* and *TP53*; Additional file 2: Figure S9).

### Mutant allele-specific expression levels vary according to mutation type, target gene, and allelic imbalance

**Fig. 3:**
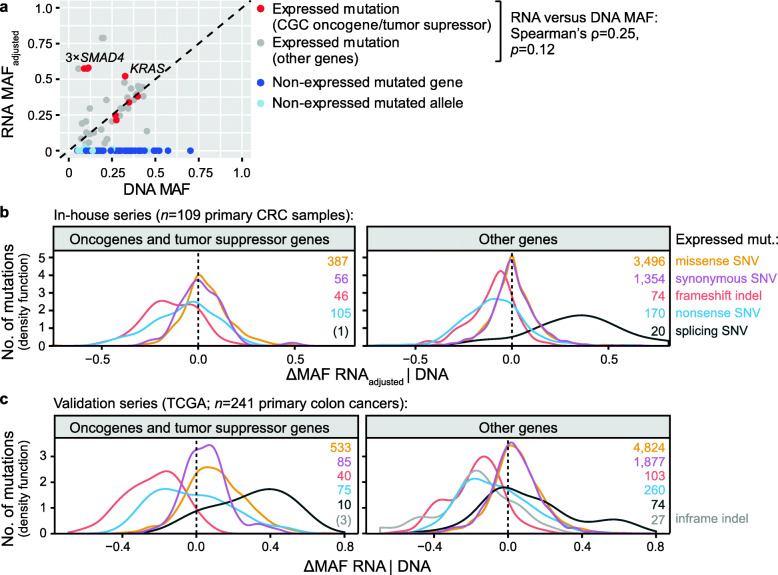
Mutant allele-specific expression levels vary by mutation type and target gene. **a** Scatter plot of MAFs in the RNA versus exome sequencing data of one selected tumor. The dashed line indicates expected expression levels according to the allelic frequency. A few target genes with preferential expression of the mutated alleles, including *SMAD* (three separate mutations) and *KRAS*, contribute to the weak statistical correlation between the RNA-level and DNA-level MAFs. The difference between the RNA-level and DNA-level MAFs (ΔMAF RNA_adjusted_|DNA) of expressed mutations in **b** the in-house tumor series and **c** the validation series from TCGA is plotted separately for mutations in oncogenes/tumor suppressor genes (as designated in the Cancer Gene Census) and other genes and grouped according to mutation types (color-coded). Only mutation types with ≥ 10 mutations are plotted, and the total numbers of mutations per mutation type are indicated. ΔMAF RNA_adjusted_|DNA above or below 0 indicate mutated loci with preferential expression of the mutated or wild-type alleles, respectively

Further investigation of preferential expression or downregulation of mutated alleles was performed after adjustment for the allelic fraction on the DNA level, evaluated as the difference between RNA MAFs and DNA MAFs (ΔMAF RNA|DNA; illustrated in Additional file 2: Figure S10). Notably, sample-wise adjustment of RNA MAFs was performed prior to analysis, since combined RNA and DNA extraction was performed for only a subset of tumors (RNA MAF_adjusted_; see the “Methods” section for the description and assessment of the adjustment). The majority of expressed mutations showed little evidence of skewed allele-specific expression levels, with RNA MAF_adjusted_ proportional to the corresponding DNA MAFs (ΔMAF RNA_adjusted_|DNA ≈ 0; illustrated for one example tumor in Fig. 3a and across all tumors in the in-house series in Additional file 2: Figure S11). The results were similar for mutations at DNA copy number balanced loci separately and after adjustment for the tumor purity of the samples (Additional file 2: Figure S11a). A separate analysis of samples with combined DNA and RNA extraction (11 samples from 6 tumors) confirmed a correlation between MAFs at the two levels (Pearson’s *r* = 0.68, *p* < 2 × 10^−16^) but also showed a minor overall skewedness towards higher RNA-level MAFs (mean sample-wise ΔMAF RNA|DNA of 0.06 [95% CI 0.02–0.09]), independent of DNA copy number imbalance and the overall expression level at the mutated locus (Pearson’s *r* = 0.04; Additional file 2: Figure S11). This indicated a slightly higher overall expression level of mutated compared to wild-type alleles, and a median of 5% (95% CI 3–20) of expressed mutations per tumor was highly overexpressed (ΔMAF RNA|DNA above 0.25). Corresponding analyses of the TCGA data supported a slight preferential expression of mutated compared to wild-type alleles (mean ΔMAF RNA|DNA 0.040 [95% CI 0.037–0.044]), again independent of allelic imbalance (Additional file 2: Figure S12). Genes with high relative allelic mutation expression (ΔMAF RNA_adjusted_|DNA > 0.25) in any tumor the in-house series are listed in Additional file 1: Table S5 and illustrated in Additional file 2: Figure S13.

Exceptions from the proportionality between RNA-level and DNA-level MAFs were found to associate with the specific mutation type and/or target gene. Expressed synonymous SNVs did not show allele-specific expression, neither in oncogenes/tumor suppressor genes nor in other genes (in-house series: mean ΔMAF RNA_adjusted_|DNA 0.01 [95% CI − 0.02–0.05] and 0.007 [95% CI 0.0009–0.01], respectively; Fig. 3b). However, truncating mutations (frameshift indels and nonsense SNVs) had reduced relative expression of the mutated allele, irrespective of the target gene category, with a mean ΔMAF RNA_adjusted_|DNA of − 0.1 both in oncogenes/tumor suppressor genes and other genes (*p* ≤ 3 × 10^−7^ in comparison with synonymous SNVs, Welch’s *t*-test). In contrast, splice site mutations had increased relative expression of the mutated allele, with a mean ΔMAF RNA_adjusted_|DNA of 0.31 (95% CI 0.20–0.42, *p* = 1 × 10^−5^ in comparison with synonymous SNVs). Missense SNV was the only mutation type with variation in allele-specific expression according to the target gene category. Missense SNVs in non-cancer-critical genes had similar relative expression to synonymous SNVs (*p* = 0.6, sample-wise paired *t*-test; Fig. 3b), while missense SNVs in oncogenes and tumor suppressor genes had slightly higher expression of the mutated allele (mean ΔMAF RNA_adjusted_|DNA of 0.04 [95% CI 0.03–0.05]), also compared to synonymous SNVs in the same set of genes (*p* = 0.01, sample-wise paired *t*-test). This skewedness was not determined by a higher overall expression level (total read count) at the mutated locus (*p* = 0.25) or by tumor purity of the samples. However, analyses according to DNA copy numbers suggested that allelic imbalance was the main determinant of higher relative allelic expression of cancer-critical missense SNVs (Additional file 2: Figure S14). The results were supported in the TCGA data (Fig. 3c), including reduced expression of truncating mutations (mean ΔMAF RNA|DNA − 0.1 both in oncogenes/tumor suppressor genes and other genes; *p* ≤ 4 × 10^−9^ in comparison with synonymous SNVs), higher expression of splice site mutations (mean ΔMAF RNA|DNA 0.14, *p* = 3 × 10^−4^), and significantly increased expression levels of missense SNVs specifically in oncogenes and tumor suppressor genes (mean ΔMAF RNA|DNA 0.1, *p* = 5 × 10^−8^, sample-wise paired *t*-test) associated with allelic imbalance at the mutated locus (missense SNVs versus synonymous SNVs: *p* = 0.8 and 0.004 at copy number balanced and unbalanced loci, respectively; Additional file 2: Figure S15). Of note, there was no difference between oncogenes and tumor suppressor genes in allelic expression levels at missense SNV loci, neither in the in-house series nor the TCGA data (*p* ≥ 0.5).

### Mutant allele-specific expression levels correlate with oncogenic signatures

**Fig. 4:**
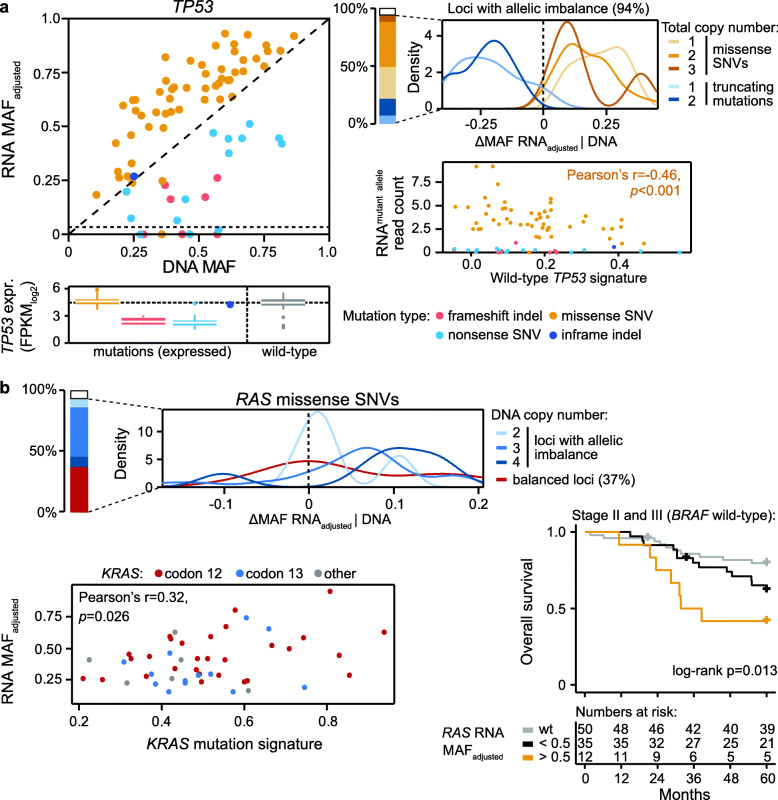
*TP53* and *RAS* mutant allele expression levels correlate with downstream oncogenic signatures. **a** Scatter plot (top left) of RNA-level versus DNA-level MAFs in *TP53*. The skewedness of mutant allele-specific expression levels according to mutation type and DNA copy numbers at the mutated loci with allelic imbalance is summarized in the density plot (top right panel) and shown to correspond to the gene expression level of *TP53* (left bottom panel). The scatter plot in the right bottom panel shows the normalized allele-specific read counts of *TP53* mutations compared to a sample-wise gene expression signature of wild-type *TP53*. The correlation is indicated for missense SNVs, while the expression levels of truncating mutations were too low for accurate analyses. **b** Density plot (top) of the difference between the RNA-level and the DNA-level MAFs in *KRAS* and *NRAS* (*RAS*) grouped according to the DNA copy number at the mutated loci (imbalanced loci with total copy number 1 or above 4 were not included in the density plot due to small group sizes). Scatter plot (bottom left) shows RNA MAFs of *KRAS* mutations (color-coded according to target codon) compared to a sample-wise gene expression signature of mutant *KRAS*. The Kaplan-Meier plot shows the 5-year overall survival in patients with *BRAF* wild-type stage II and III CRC, grouped according to *RAS* mutation status (wt, wild-type) and RNA MAF_adjusted_ of the missense SNVs

Focused analyses of the three genes with the most frequent mutation expression further illustrated the relationship among mutation types, allelic imbalance, and allelic expression levels. *TP53* was affected by both missense SNVs and putative truncating mutations, most of which also had allelic imbalance (94% of all *TP53* mutations). Missense SNVs had significantly higher allele-specific expression than putative truncating mutations (in-house series: mean ΔMAF RNA_adjusted_|DNA 0.18 and − 0.23, respectively, *p* = 4 × 10^−12^, Welch’s *t*-test), independent of the total DNA copy number at the mutated locus (Fig. 4a). The allelic expression patterns further corresponded with the overall gene expression levels of *TP53*, and tumors with truncating mutations had lower *TP53* expression than tumors with either missense SNVs or wild-type *TP53* (*p* < 1 × 10^−9^). A downstream functional impact was suggested by an inverse correlation between the expression of missense SNVs (analyzed as the normalized RNA read count of mutated alleles) and a sample-wise gene expression signature of wild-type *TP53* (Pearson’s *r* = − 0.46, *p* < 0.001). Validation analyses in the TCGA data supported the allele-specific mutation expression patterns, including the inverse association with the *TP53* expression signature (*p* = 0.02; Additional file 2: Figure S16a), suggesting that the downstream impact of TP53 in MSS CRC is regulated by a complex targeting of the gene, involving both mutations and allelic imbalance.

The *KRAS* and *NRAS* (*RAS*) oncogenes were targeted by missense SNVs only, and their allele-specific expression (RNA MAF_adjusted_) was generally proportional to the corresponding DNA MAFs (in-house series: Pearson’s *r* = 0.89, *p* = 2 × 10^−16^), although the ΔMAF RNA_adjusted_|DNA was slightly higher at loci with allelic imbalance caused by copy number gain (Fig. 4b). The relative expression levels of *KRAS* mutant alleles showed a weak but significant correlation with a sample-wise oncogenic KRAS signature, both in the in-house series (Pearson’s *r* = 0.32, *p* = 0.026) and the TCGA data (Pearson’s *r* = 0.43, *p* = 4 × 10^−5^; Additional file 2: Figure S16b), suggesting that high relative allelic expression of the mutations increases oncogenic KRAS signaling. Consistently, high allelic expression of *RAS* mutations was also associated with poorer 5-year overall survival among patients with stage II and III cancers (including only patients treated by complete resection for *BRAF* wild-type tumors; Fig. 4b). The univariate and multivariable hazard ratios for *RAS* RNA MAF_adjusted_ as a continuous variable were 6.1 and 4.6, respectively (Wald test *p* = 0.0070 and 0.042; the multivariable model included parameters listed in Table 1).

Mutant allele expression levels of *APC* were dependent on two main factors, the position of the mutation and the presence of double mutations. Using truncating nonsense SNVs for illustration, mutations affecting the 3′ region of the gene had higher allele-specific expression than more upstream mutations (*p* = 2 × 10^−8^; Additional file 2: Figure S17a), consistent with activation of nonsense-mediated mRNA decay (NMD) only by the latter group [43]. Furthermore, targeting of *APC* by two mutations appeared to have less effect than single mutations, and tumors with double mutations had a significantly lower both DNA-level and RNA-level MAF (*p* < 1 × 10^−3^ by Welch’s *t*-test). Only single truncating mutations were associated with a lower *APC* gene expression level compared to *APC* wild-type tumors (Additional file 2: Figure S17b).

### High allele-specific expression of resistance mutations may negatively impact sensitivity to targeted anticancer agents

**Fig. 5:**
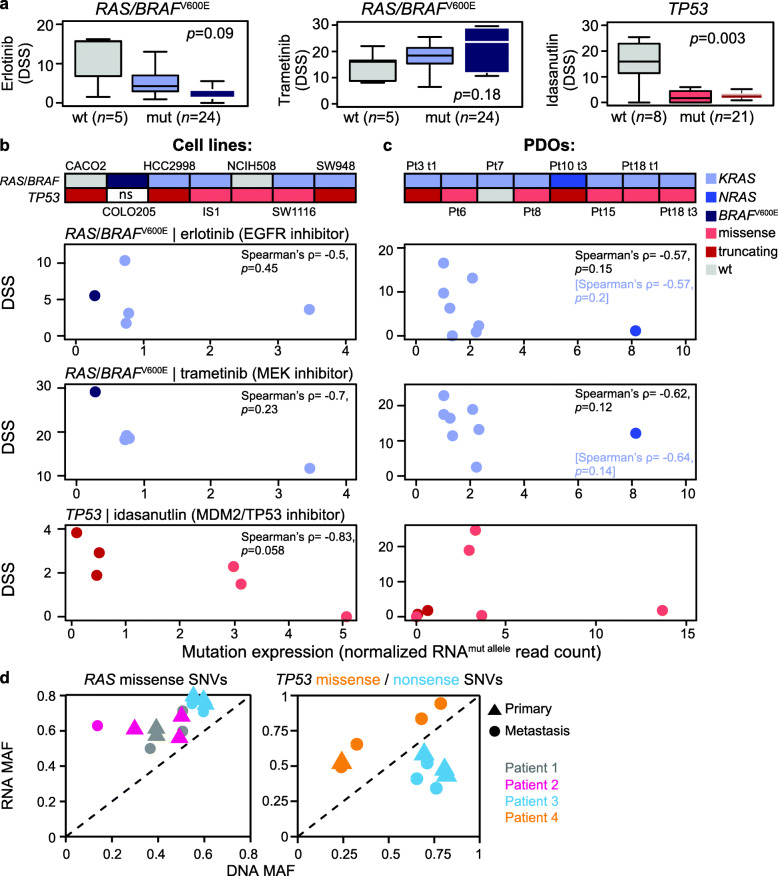
Correlation between *TP53* and *RAS*/*BRAF*^V600E^ mutation expression levels and sensitivity to targeted anticancer agents in pre-clinical models. **a** Sensitivity to the EGFR inhibitor erlotinib, the MEK inhibitor trametinib, and the MDM2 inhibitor idasanutlin in a panel of 29 unique CRC cell lines plotted according to *RAS*/*BRAF*^V600E^ or *TP53* mutation status, as indicated (mut, mutated; wt, wild-type; color codes are shown in **c**). Higher DSS indicates stronger sensitivity. *p*-value is from Welch’s *t*-test of wild-type versus mutated samples. **b**, **c** Upper panels show the mutation status for *RAS*/*BRAF*^V600E^ and *TP53* in each of the 7 selected cell lines and 8 patient-derived organoids (PDOs). Scatter plots show the DSS of matched drugs versus mutant allele expression levels (color-coded as indicated). Spearman’s correlations in blue are for *KRAS*-mutated PDOs only (excluding the single *NRAS*-mutated sample). **d** Scatter plot of RNA-level versus DNA-level MAFs of *RAS* and *TP53* in matched primary and metastatic tumor samples from each of four patients (three with *RAS* mutations and two with *TP53* mutations). Patient 2 showed higher relative expression of the *RAS* mutant allele in the metastasis

To further investigate a potential functional impact of allelic *TP53* and *RAS*/*BRAF* mutation expression levels, sensitivity to relevant anticancer agents was analyzed in pre-clinical models. Across a panel of 29 unique CRC cell lines (Additional file 1: Table S6), samples with *RAS*/*BRAF*^V600E^ or *TP53* mutations had low sensitivity to erlotinib (EGFR inhibitor) and idasanutlin (MDM2/TP53 inhibitor), respectively, while no association between *RAS*/*BRAF*^V600E^ mutation status and sensitivity to trametinib (MEK inhibitor) was found (Fig. 5a). Notably, the variation in sensitivity to erlotinib among cell lines with *RAS*/*BRAF*^V600E^ mutations (10–90th percentile of DSS values 1.3–9.9) was much larger than for idasanutlin among samples with *TP53* mutations (10–90th percentile 0–5.1), reflected also in a weaker statistical difference between the *RAS*/*BRAF*^V600E^ mutated and wild-type groups. There was no significant difference in sensitivity to idasanutlin between samples with truncating and missense mutations in *TP53* (*p* = 0.25; Additional file 2: Figure S18).

Seven of the cell lines were selected for RNA sequencing based on molecular characteristics (MSS, *RAS*/*BRAF*^V600E^, and/or *TP53* mutations) and a large range in sensitivity to the three drugs (Additional file 1: Table S6). There were indications of a negative correlation between the allelic expression levels (normalized allele-specific read counts) of *RAS*/*BRAF*^V600E^ mutations and sensitivity to erlotinib and trametinib, as well as between *TP53* mutations and sensitivity to idasanutlin, although not statistically significant in this small sample set (Fig. 5b). The relationship was strongest for *TP53* mutations and idasanutlin (Spearman’s *ρ* = − 0.83, *p* = 0.058). For independent validation, eight PDOs from resected MSS CRC liver metastases were similarly selected and analyzed (Additional file 1: Table S7). Two individual PDOs with particularly high allele-specific expression levels of an *NRAS* or a *TP53* mutation showed strong resistance to erlotinib and idasanutlin, respectively. This supported the negative impact of a high mutant allele-specific expression on drug sensitivity for both gene-drug pairs, and the correlation was strongest for *RAS*|erlotinib (Spearman’s *ρ* = − 0.57, *p* = 0.15, not statistically significant), also when analyzing *KRAS* mutated PDOs only (Fig. 5c). Notably, there is no established relationship between *RAS*/*BRAF*^V600E^ mutations and sensitivity to MEK inhibition, but our data suggested a negative correlation with allelic mutation expression levels, which among PDOs was associated with a sample-wise correlation in MEK and EGFR inhibitor sensitivity (Pearson’s *r* = 0.85, *p* = 0.007).

Targeted therapies are primarily used in the metastatic setting in CRC, and a comparison of matched primary and metastatic tumors from 4 patients (*n* = 4–6 samples per patient) showed a strong proportionality in the patterns of allelic expression of mutations between the two disease settings (Additional file 2: Figure S19 and Figures S20-S23). This included a significantly higher mean ΔMAF RNA|DNA of missense SNVs in oncogenes and tumor suppressor compared to other genes also in metastases (*p* = 0.008), independent of previous exposure to chemotherapy (none of the patients received targeted therapy prior to sampling). Proportional allelic expression levels were found also for *RAS* and *TP53* mutations, although one of the three patients with *RAS* mutations had higher relative expression of the mutated allele in the metastasis (ΔMAF RNA|DNA 0.49) than the primary tumor samples (range 0.07–0.31; Fig. 5d), suggesting selection for addiction to KRAS signaling and resistance to anti-EGFR therapy during metastasis of this cancer.

## Discussion

The “gene dosage” effect of DNA copy number aberrations explains the association between *ERBB2* amplification, HER2 protein over-expression, and response to HER2 targeted combination therapies in metastatic CRC [44]. Similarly, “mutation dosage” analyzed as the allelic frequency of *RAS*/*BRAF*^V600E^/*PIK3CA* mutations has been shown to inversely correlate with response to anti-EGFR therapy [8]. In this study, we followed the same reasoning and analyzed “mutation dosage” on the RNA expression level. In light of the large efforts to map the mutational landscape of CRCs [9, 45–47], surprisingly, few studies have evaluated mutations at the expression level. Our study supported the power of integrated genomic and transcriptomic profiling. Firstly, the majority of mutations in the coding regions of CRC genomes were not expressed (found in non-expressed genes). In contrast, the majority of mutations in well-known CRC-critical genes were expressed. Secondly, there was strong, overall proportionality between the allelic frequencies and allele-specific expression levels of mutations across this relatively large MSS CRC series. However, there were also indications of more frequent expression of mutated compared to corresponding wild-type alleles in general, independent of allelic imbalance. This overall skewedness was small and context-dependent. As an illustrative example, *TP53* had significantly reduced allele-specific expression of putative truncating mutations, but preferential expression of the mutated alleles at missense SNV loci. The latter is in line with published results for *TP53* mutations in other cancer types [16, 48, 49], and our study suggested an association both with downstream TP53 transcriptional activity and combined genetic targeting by allelic imbalance.

Low expression levels of putative truncating mutations (nonsense SNVs and frameshift indels) are likely explained by NMD and degradation of transcripts with premature termination codons, as previously shown in breast cancers [12] and cancer cell lines [17]. This association was particularly clear for nonsense SNVs in *APC*. NMD is not activated against premature termination codons in the 3′ end of transcripts [43], and only nonsense SNVs occurring closer to the 5′ end of *APC* had reduced allele-specific expression. Furthermore, the observed preferential expression of mutant alleles at splice sites is consistent with the failure of the splicing machinery to recognize these sites. Splice site-disrupting mutations may therefore have higher RNA levels as a result of aberrant intron retention, although intron retention has been recognized as a widespread mechanism for tumor suppressor inactivation, caused by NMD acting on premature termination codons commonly located in introns [50]. These biological mechanisms support a deterministic role of the mutation category on allele-specific expression levels. In contrast, exonic missense SNV expression was further dependent on the target gene category, with higher relative expression of mutated alleles in cancer-critical genes specifically, associated with the previously reported selection for oncogenic allelic imbalance in cancer [51]. Notably, there was no consistent difference between oncogenes and tumor suppressor genes with respect to allelic expression levels. Based on the expectation that tumor suppressor mutations act by loss of function, this suggests that inactivation occurs at the level of protein expression and/or modification. It should be noted that these genome-wide observations may conceal gene-specific features, and the low number of prevalently mutated genes in MSS CRCs precluded more detailed analyses. Indeed, a gene-specific feature was identified in *APC*, which is a gene commonly targeted by more than one mutation. The seemingly lower effect of *APC* mutations in double-targeted tumors is interesting in light of the reported difference in prognostic associations of single and double-targeted MSS CRCs, although the single-mutated group had improved survival compared to double-mutated and *APC* wild-type cancers [52].

Proof-of-concept analyses of a functional consequence of allele-specific mutation expression levels were performed by evaluation of potential predictive value for sensitivity to targeted anticancer agents in pre-clinical models. The well-known associations between the mutation status of *RAS*/*BRAF*^V600^ or *TP53* and sensitivity to EGFR or MDM2 inhibition, respectively, were accompanied by variation in the level of drug resistance among mutated samples, in particular for EGFR inhibition. Our study suggested a fine-tuned association with the expression level of the corresponding resistance mutation. This is consistent with clinical data demonstrating the efficacy of rechallenge with anti-EGFR therapies after initial progression on treatment, when guided by *RAS* mutation levels in the blood [53]. However, care should be taken in the interpretation of these data due to the small sample size. Furthermore, other gene mutations with a potential influence on drug sensitivity were not controlled for, such as additional resistance factors for EGFR inhibition in the MAPK signaling pathway [53]. It should also be noted that MDM2 inhibition guided by wild-type *TP53* is not a clinically validated treatment strategy for patients with CRC, and the rationale for this analysis was based on previously published pre-clinical data [54]. Finally, the pharmacogenomic analyses did not allow discrimination between allelic fractions at the DNA and RNA levels. It has been shown that sensitivity to MAPK inhibition in CRC cell lines increases with the allelic frequency of *KRAS* mutations [55]. Furthermore, allelic imbalance at mutated *KRAS* loci may be associated with poor patient survival in CRC, compared to tumors with balanced mutated loci [51]. In our study, poor prognostic associations of a high *RAS* MAF were also found at the RNA level, and the relative allelic mutation expression was indeed higher at loci with allelic imbalance and copy number gain. This suggests that the genomic aberrations are faithfully recapitulated at the expression level, mediating a mutation-associated gain-of-fitness to the cancer cells. Comparisons of patient-matched primary and metastatic tumors also suggested proportionality in allelic mutation expression levels during metastasis. However, based on the current study, we foresee that the expression levels of “actionable” mutations will be highly variable in response to targeted agents.

## Conclusions

This study reports the first large-scale analysis of allele-specific mutation expression in CRC and indicated an opportunity for more fine-tuned biomarker interpretations. Analyses in relation to oncogenic signatures, patient survival, and targeted drug sensitivity in pre-clinical models proposed that the “expressed mutation dose” has functional consequences.

## Supplementary Information

**Additional file 1: Table S1.** Clinicopathological characteristics and expressed tumor mutational burden in primary colon cancers from TCGA. **Table S2.** Summarization of the different mutation categories detected by whole-exome sequencing of 126 samples from 121 microsatellite stable CRCs (in-house series). **Table S3.** Filtering of mutations for allele-specific expression analysis based on RNA sequencing coverage. **Table S4.** Mutation expression frequency. **Table S5.** Non-synonymous mutations with high relative allelic expression level (ΔMAF RNA_adjusted_|DNA > 0.25). **Table S6.** Overview of CRC cell lines (*n* = 29). **Table S7.** Overview of PDOs from resected microsatellite stable CRC liver metastases analyzed by RNA sequencing and drug sensitivity testing.

**Additional file 2: Figure S1.** TMB and expressed TMB relative to sequencing coverage and tumor purity. **Figure S2.** Gene-wise mutation frequency among microsatellite stable CRCs. **Figure S3.** Gene set enrichment analyses of expressed mutations. **Figure S4.** Validation of mutation expression frequencies in TCGA. **Figure S5.** Heatmap of expressed mutations per gene and tumor sample. **Figure S6.** Logistic PCA of the expressed mutation matrix. **Figure S7.** Genes with frequent non-expressed mutations. **Figure S8.** Genes with frequent non-expressed mutated alleles. **Figure S9.** Genes with both expression and silencing of the mutated allele among tumors. **Figure S10.** Illustration of estimates used to describe relative allelic expression of mutations. **Figure S11.** RNA and DNA level MAFs in primary MSS CRCs in the in-house series. **Figure S12.** RNA MAF *versus* DNA MAF in TCGA. **Figure S13.** Genes with high relative allelic expression of mutations. **Figure S14.** RNA MAF *versus* DNA MAF in in-house series according to mutation/target gene category. **Figure S15.** Relative allelic mutation expression in TCGA data according to allelic copy number balance. **Figure S16.** Allele-specific expression of *TP53* and *KRAS* mutations among 241 primary colon cancers in the TCGA validation series. **Figure S17.** Allele-specific expression of *APC* mutations in the in-house series of primary CRCs. **Figure S18.** Allele-specific mutation expression and drug sensitivity in CRC cell lines. **Figure S19.** Minimal evolutionary changes in mutation expression during metastasis. **Figure S20.** Comparisons of DNA MAFs among multiple tumor samples from each of 4 patients. **Figure S21.** Comparisons of RNA MAFs among multiple tumor samples from each of 4 patients. **Figure S22.** Comparisons of ΔMAF RNA|DNA among multiple tumor samples from each of 4 patients. **Figure S23.** Comparisons of RNA read counts of mutated alleles among multiple tumor samples from each of 4 patients.

## Data Availability

In accordance with Norwegian legislation and the ethical approval of the study by the Regional Committee for Medical and Health Research Ethics, South-Eastern Norway, the raw high-throughput DNA and RNA sequencing data generated in this study are considered patient identifiable and subject to secure storage regulations in accordance with the national Personal Data Regulations, chapter 2. Data can currently not be deposited into public repositories. Data will be made available upon reasonable request to the corresponding author, and this will require the formalization of a data transfer agreement.

## Abbreviations

ΔMAF RNA|DNA: Difference between the RNA-level and DNA-level MAF
CI: Confidence interval
CGC: Cancer Gene Census
CRC: Colorectal cancer
DSS: Drug sensitivity score
FPKM: Fragments per kilobase of transcripts per million mapped reads
indel: Insertions and deletions
MAF: Mutant allele fraction
MSS: Microsatellite stable
NMD: Nonsense-mediated mRNA decay
PCA: Principal component analysis
PDO: Patient-derived tumor organoid
*RAS*: *KRAS*/*NRAS*
SNV: Single nucleotide variant
TCGA: The Cancer Genome Atlas
TMB: Tumor mutational burden
