# Supplementary material for: The expressed mutational landscape of microsatellite stable colorectal cancers
Source: Genome Med. 2021 Sep 1;13:142. doi: 10.1186/s13073-021-00955-2 (PMC8411524; doi:10.1186/s13073-021-00955-2)
Supplement: Supplementary file 2 — Additional file 2: Figure S1. TMB and expressed TMB relative to sequencing coverage and tumor purity. Figure S2. Gene-wise mutation frequency among microsatellite stable CRCs. Figure S3. Gene set enrichment analyses of expressed mutations. Figure S4. Validation of mutation expression frequencies in TCGA. Figure S5. Heatmap of expressed mutations per gene and tumor sample. Figure S6. Logistic PCA of the expressed mutation matrix. Figure S7. Genes with frequent non-expressed mutations. Figure S8. Genes with frequent non-expressed mutated alleles. Figure S9. Genes with both expression and silencing of the mutated allele among tumors. Figure S10. Illustration of estimates used to describe relative allelic expression of mutations. Figure S11. RNA and DNA level MAFs in primary MSS CRCs in the in-house series. Figure S12. RNA MAF versus DNA MAF in TCGA. Figure S13. Genes with high relative allelic expression of mutations. Figure S14. RNA MAF versus DNA MAF in in-house series according to mutation/target gene category. Figure S15. Relative allelic mutation expression in TCGA data according to allelic copy number balance. Figure S16. Allele-specific expression of TP53 and KRAS mutations among 241 primary colon cancers in the TCGA validation series. Figure S17. Allele-specific expression of APC mutations in the in-house series of primary CRCs. Figure S18. Allele-specific mutation expression and drug sensitivity in CRC cell lines. Figure S19. Minimal evolutionary changes in mutation expression during metastasis. Figure S20. Comparisons of DNA MAFs among multiple tumor samples from each of 4 patients. Figure S21. Comparisons of RNA MAFs among multiple tumor samples from each of 4 patients. Figure S22. Comparisons of ΔMAF RNA|DNA among multiple tumor samples from each of 4 patients. Figure S23. Comparisons of RNA read counts of mutated alleles among multiple tumor samples from each of 4 patients. [file 13073_2021_955_MOESM2_ESM.pdf]

Additional file 2: Supplementary Figures S1 to S23 for

The expressed mutational landscape of microsatellite stable colorectal cancers

Anita Sveen, Bjarne Johannessen, Ina A. Eilertsen, Bård I. Røsok, Marie Gulla, Peter W. Eide, Jarle Bruun, Kushtrim Kryeziu, Leonardo A. Meza-Zepeda, Ola Myklebost, Bjørn A. Bjørnbeth, Rolf I. Skotheim, Arild Nesbakken, Ragnhild A. Lothe

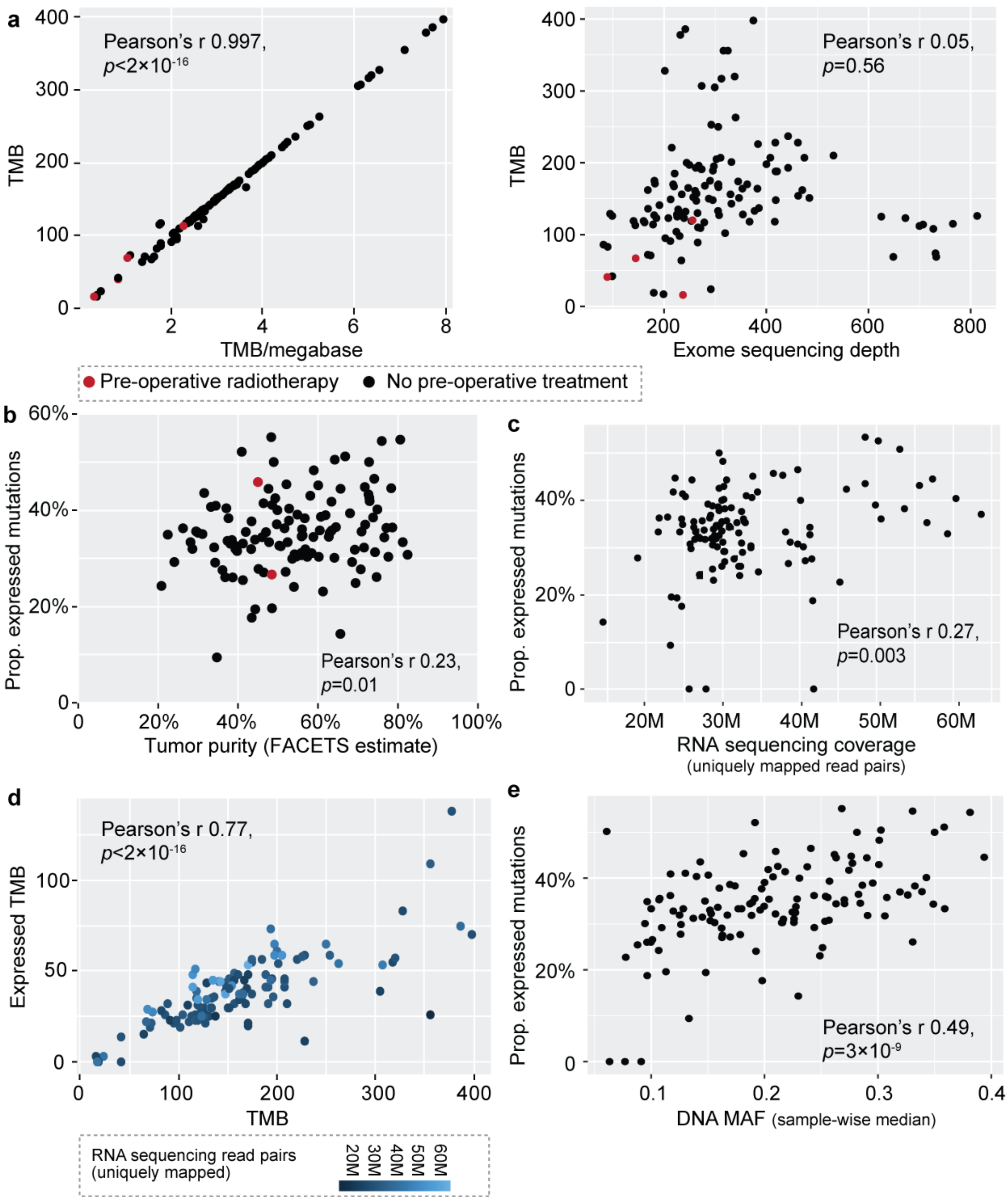

Figure S1. TMB and expressed TMB relative to sequencing coverage and tumor purity

a) The TMB was highly correlated to the number of nonsynonymous and frameshift mutations per sequenced megabase (TMB/megabase; left) and was not dependent on the exome sequencing depth (right). The proportion of expressed mutations per tumor was weakly associated with **b)** the tumor purity (estimated by FACETS based on DNA copy numbers; non-available for 7 samples), and **c)** the RNA sequencing depth, plotted as the number of uniquely mapped read pairs per tumor. **d)** However, the number of expressed mutations was much stronger correlated with the TMB. The 3 tumors with no expressed mutations had both a low TMB and **(e)** low mutant allele fractions on the DNA level (DNA MAF). Each dot represents one tumor sample.

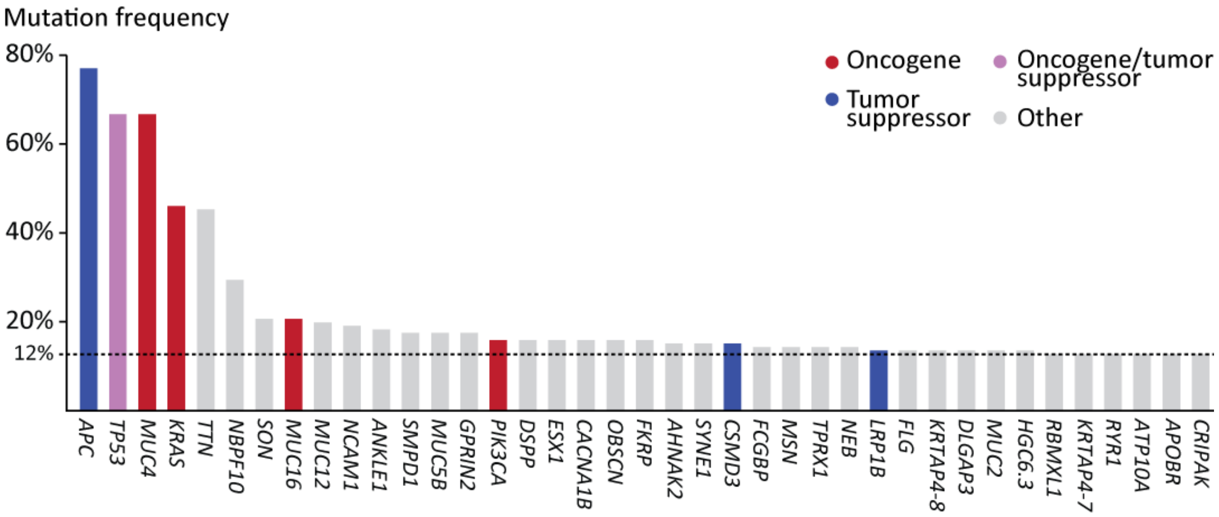

Figure S2. Gene-wise mutation frequency among microsatellite stable CRCs

Genes (n = 39) with nonsynonymous or frameshift mutations in more than 12% of the 126 primary CRC samples, detected by whole exome sequencing. Genes are ordered according to decreasing mutation frequency, and colored according to gene category, as defined in the Cancer Gene Census.

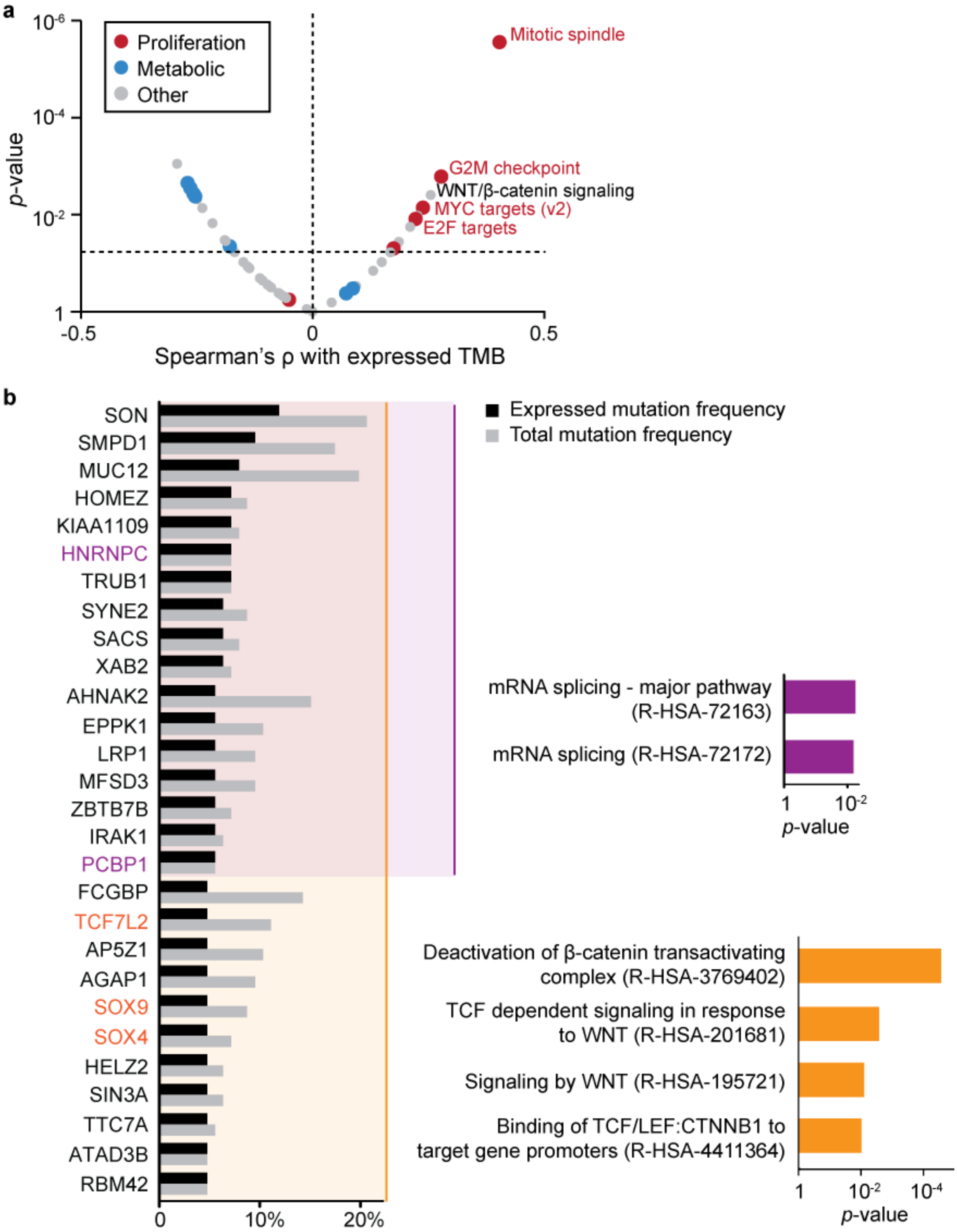

Figure S3. Gene set enrichment analyses of expressed mutations

a) Correlation analysis (plotted as Spearman's correlation coefficient and corresponding *p*-value) between single-sample gene set scores of each gene set in the hallmark collection (n = 50) and the expressed TMB of 126 tumors samples in the in-house series. Selected categories of gene sets (red and blue) were as denoted in Liberzon et al. *Cell Syst* 2015, **1**:417-425. **b)** Bar plot to the left shows genes (excluding oncogenes and/or tumor suppressors) with the most frequent, expressed mutations. Barplots to the right show results from enrichment analysis of the Reactome pathway database (using the Enrichr web server), based on the top-17 genes (purple) and all 28 genes (orange). Genes contained within the gene sets with significant enrichment are indicated by the corresponding color in the left bar plot.

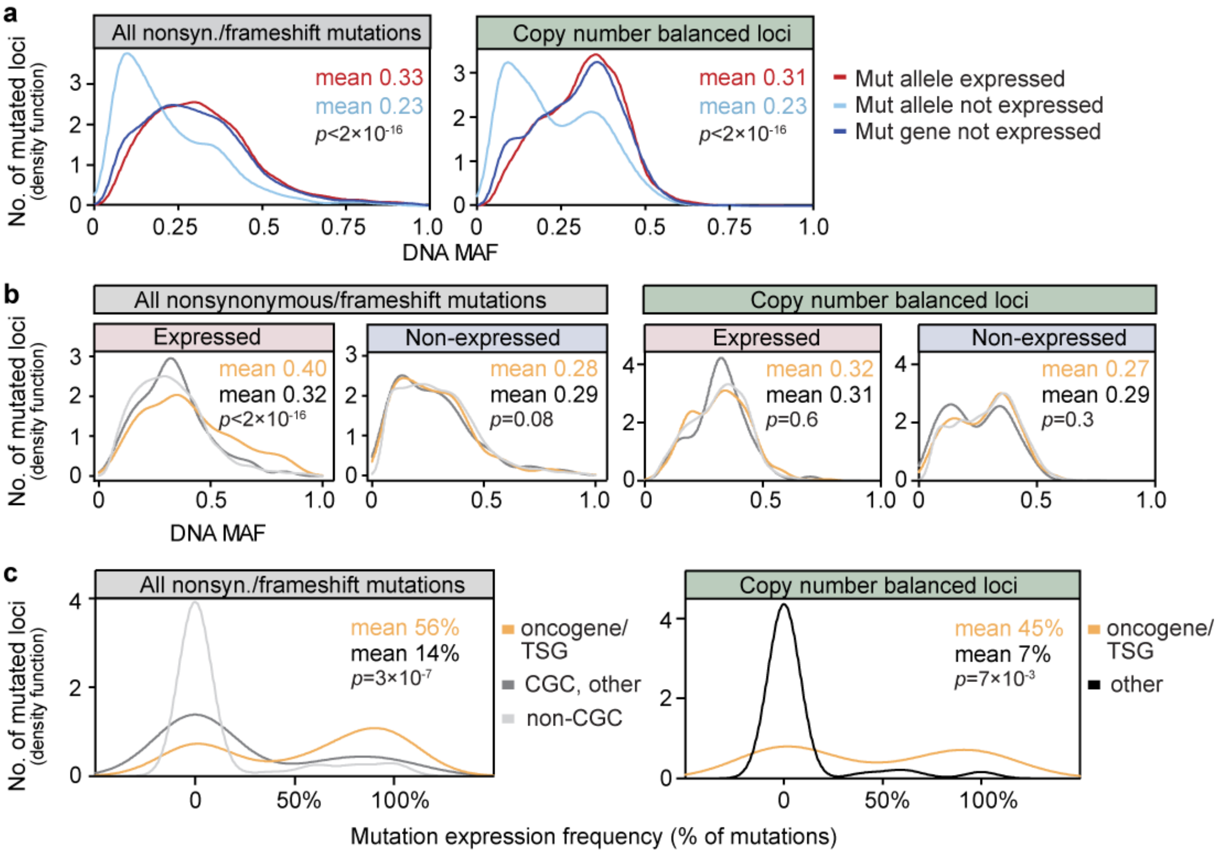

Figure S4. Validation of mutation expression frequencies in TCGA

a) Validation analyses performed in 241 MSS colon cancers from TCGA supported that mutated loci with wild-type only expression had lower DNA MAFs than loci with expression of the mutated allele (left), also at loci with balanced DNA copy numbers (equal number of copies of the mutated and wild-type alleles; right). **b)** The higher DNA MAF of expressed mutations in oncogenes and tumor suppressor genes (compared both to expressed mutations in other genes and all non-expressed mutations) was not found at loci with balanced copy numbers, indicating that this is associated with allelic imbalance. **c)** Nonetheless, the more frequent expression of mutations in oncogenes and tumor suppressor genes was independent of allelic imbalance. In b-c) the mean is reported for mutations in oncogenes/tumor suppressor genes (yellow) and all other genes (black) separately. In c), only genes mutated in ≥5 samples each are included.

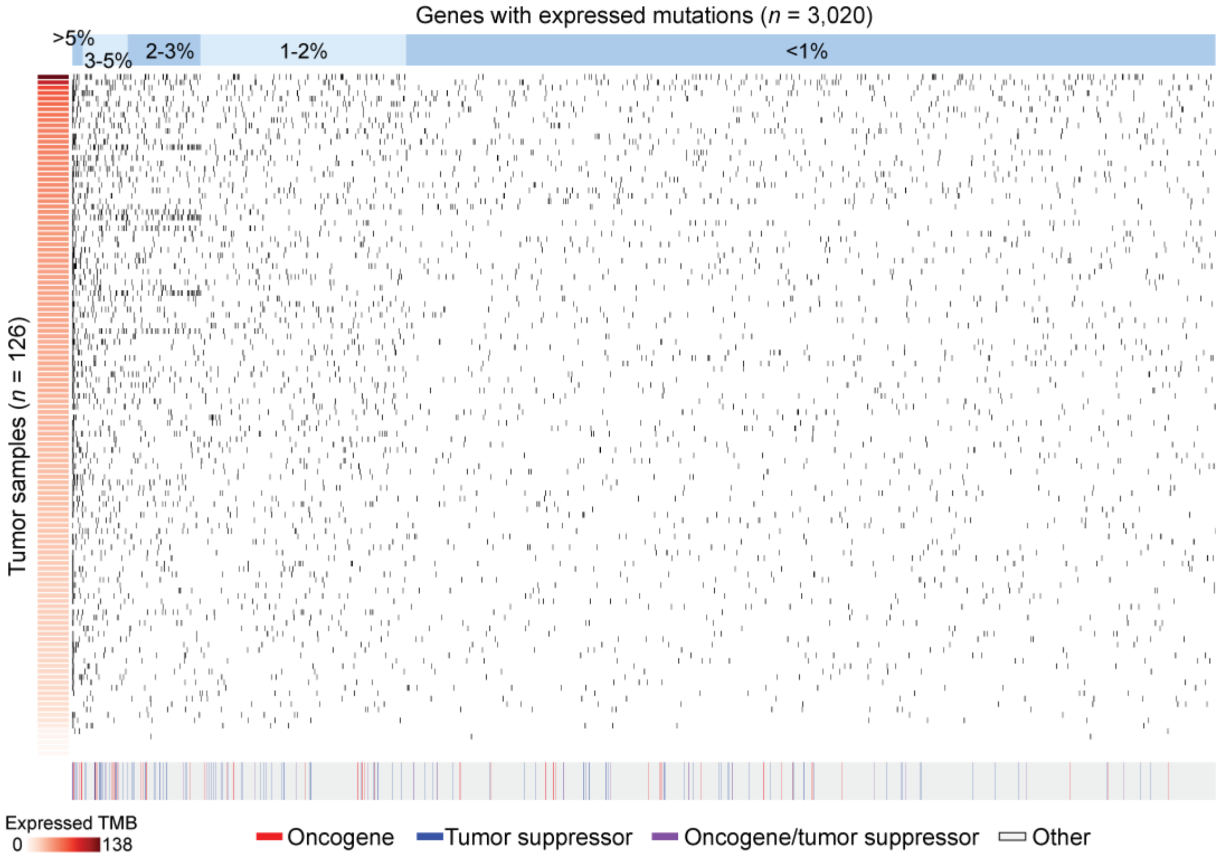

Figure S5. Heatmap of expressed mutations per gene and tumor sample

Black bars in the heatmap indicate a sample and gene with an expressed mutation. Each row represents a tumor sample, and each column represents a gene with an expressed mutation. Samples are ordered according to their expressed TMB (left bar), and genes are ordered according to their mutation expression frequency (proportion of tumors with an expressed mutation; top bar). Bottom bar shows the target gene category as indicated (according to the Cancer Gene Census).

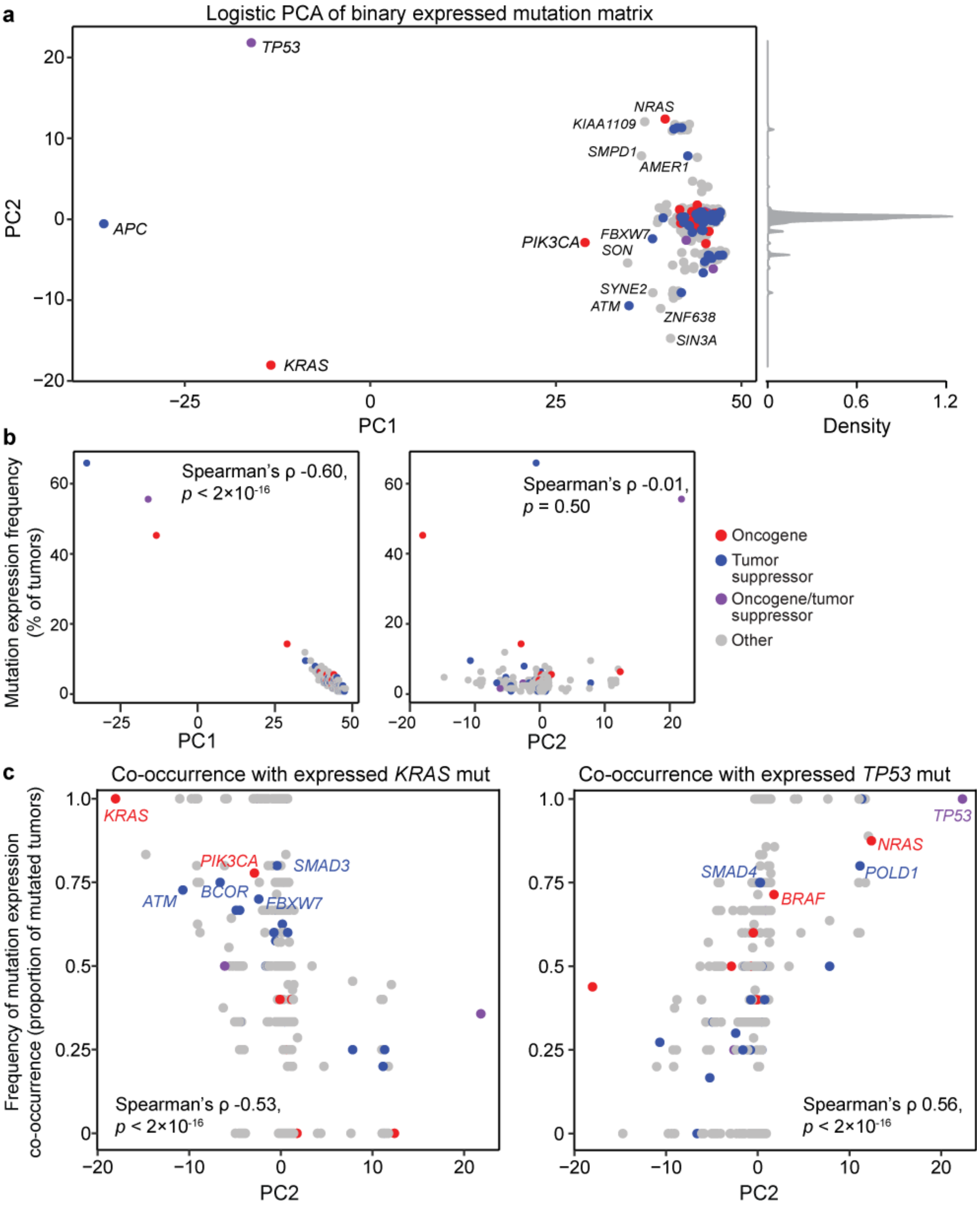

Figure S6. Logistic PCA of the expressed mutation matrix

a) PCA plot shows results from binary dimensionality reduction of genes with expressed mutations, based on logistic PCA of the sample- and gene-wise expressed mutation matrix illustrated in Figure S5. Indicated to the right is the gene density along PC2. **b)** Scatter plots of the mutation expression frequency of each gene (proportion of tumors with an expressed mutation) *versus* PC1 (left) and PC2 (right) from logistic PCA. **c)** Scatter plots of genes with expressed mutations co-occurring with expressed mutations in *KRAS* (left) and *TP53* (right), plotted as the proportion of expressed mutations *versus* PC2 from logistic PCA.

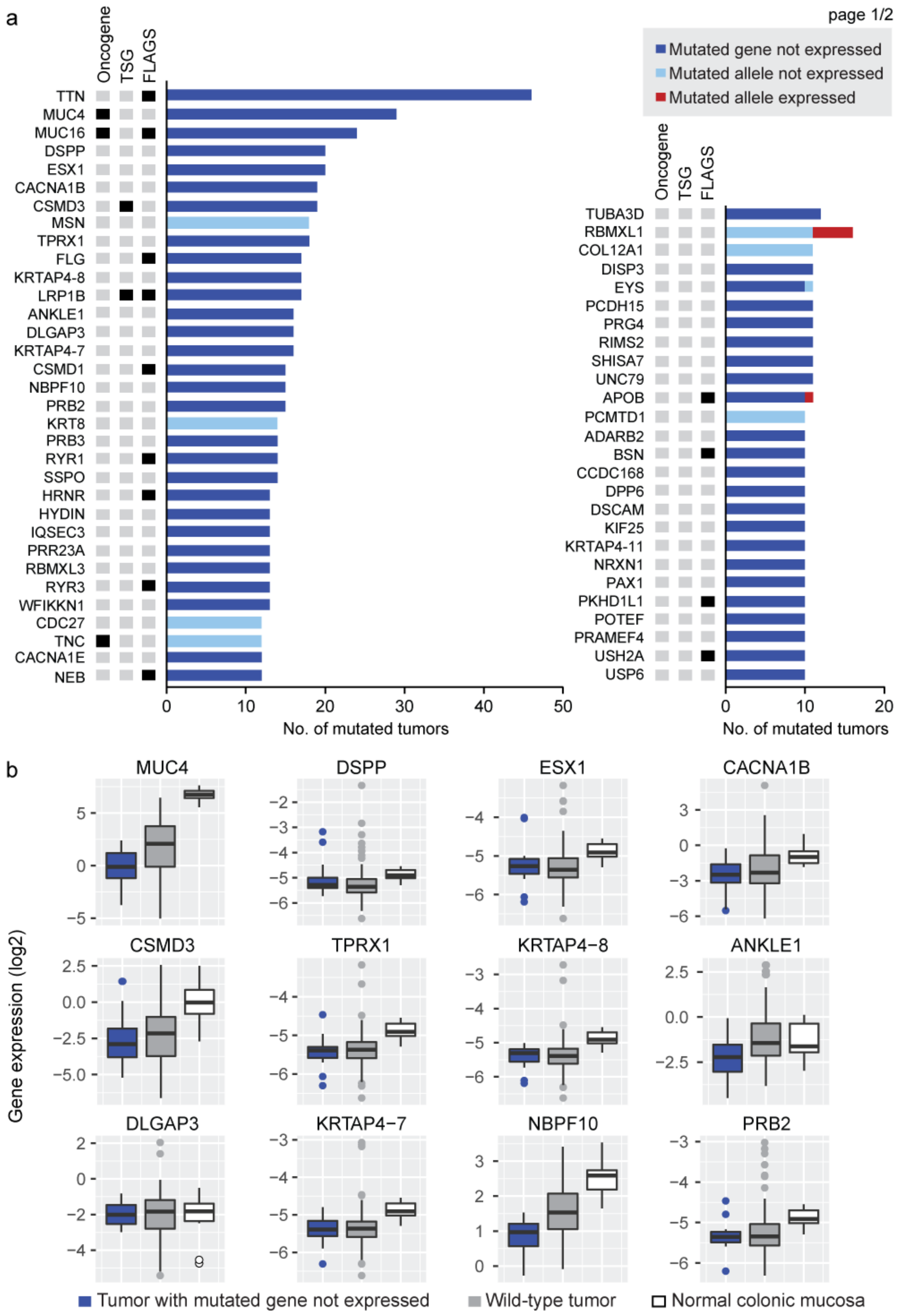
a
Oncogene
TSG
FLAGS
Mutated gene not expressed
Mutated allele not expressed
Mutated allele expressed
TTN
MUC4
MUC16
DSPP
ESX1
CACNA1B
CSMD3
MSN
TPRX1
FLG
KRTAP4-8
LRP1B
ANKLE1
DLGAP3
KRTAP4-7
CSMD1
NBPF10
PRB2
KRT8
PRB3
RYR1
SSPO
HRNR
HYDIN
IQSEC3
PRR23A
RBMXL3
RYR3
WFIKKN1
CDC27
TNC
CACNA1E
NEB
TUBA3D
RBMXL1
COL12A1
DISP3
EYS
PCDH15
PRG4
RIMS2
SHISA7
UNC79
APOB
PCMTD1
ADARB2
BSN
CCDC168
DPP6
DSCAM
KIF25
KRTAP4-11
NRXN1
PAX1
PKHD1L1
POTEF
PRAMEF4
USH2A
USP6
No. of mutated tumors
b
MUC4
DSPP
ESX1
CACNA1B
CSMD3
TPRX1
KRTAP4−8
ANKLE1
DLGAP3
KRTAP4−7
NBPF10
PRB2
Gene expression (log2)
Tumor with mutated gene not expressed
Wild-type tumor
Normal colonic mucosa

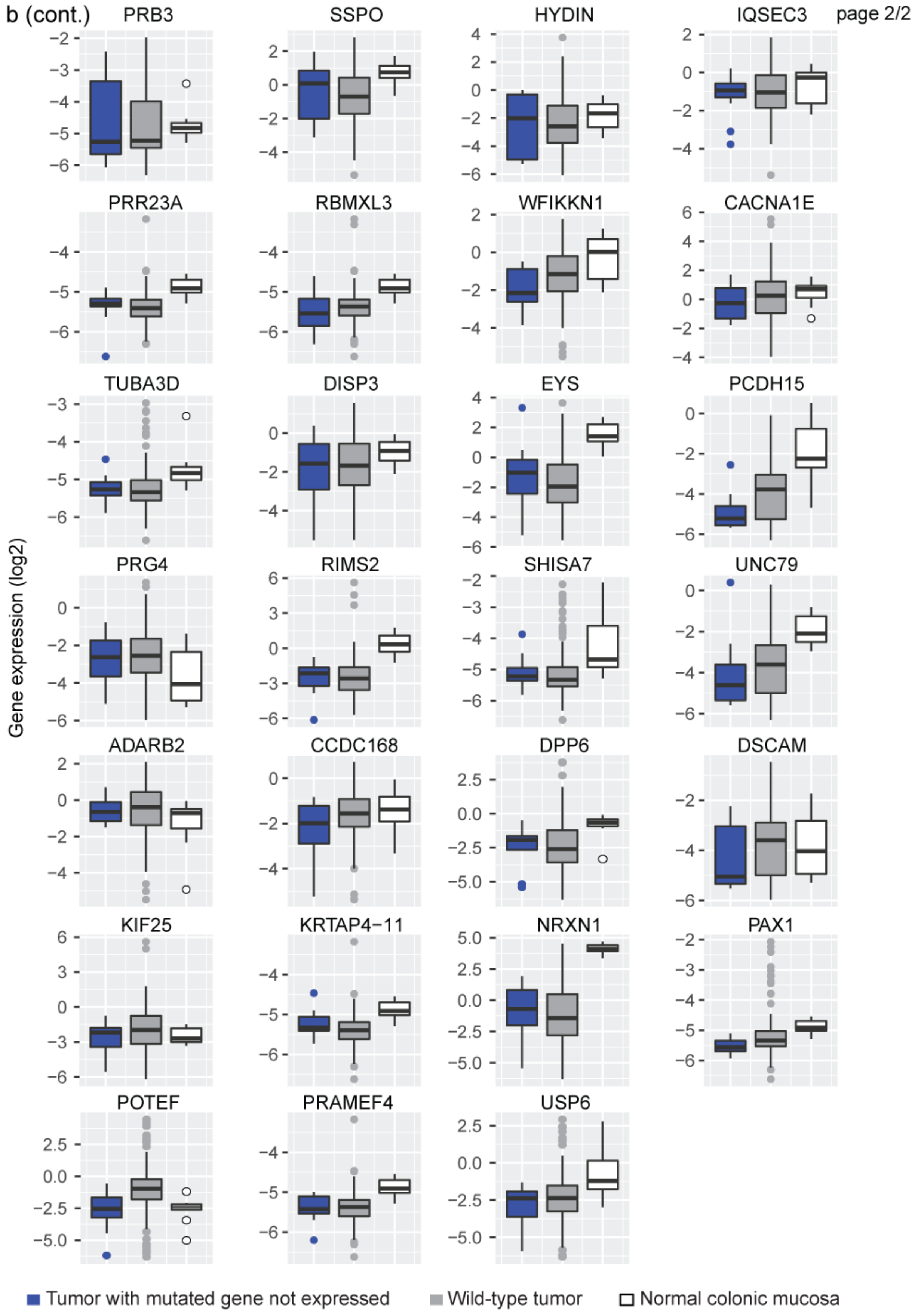

b (cont.)
page 2/2
PRB3
SSPO
HYDIN
IQSEC3
PRR23A
RBMXL3
WFIKKN1
CACNA1E
TUBA3D
DISP3
EYS
PCDH15
PRG4
RIMS2
SHISA7
UNC79
ADARB2
CCDC168
DPP6
DSCAM
KIF25
KRTAP4−11
NRXN1
PAX1
POTEF
PRAMEF4
USP6
Gene expression (log2)
Tumor with mutated gene not expressed
Wild-type tumor
Normal colonic mucosa

Figure S7. Genes with frequent non-expressed mutations

a) Stacked bar plot shows all genes (*n* = 59) with non-expressed mutations in ≥10 tumors (sorted by the number of mutated tumors in which the gene is not expressed, and divided into two columns for plotting purposes). Panels to the left indicate genes (black squares) that are denoted as oncogenes or tumor suppressor genes (TSG) in the CGC, and/or as FLAGS in Shyr et al., *BMC Med Genomics* 2014, **7:**64. Categorization of non-expressed genes (dark blue) was based on zFPKM transformation (Methods). Only mutated loci with sufficient coverage in the RNA sequencing data were included for analysis (Methods). **b)** Boxplots of gene expression levels (log2-scale; normalized HTSeq-count data by the trimmed mean of M values and voom-transformation) across tumors (categorized according to mutation status for the respective gene) and normal colonic mucosa samples (*n* = 12). Included are all genes (*n* = 39) that were non-expressed in tumors in which the gene was mutated (indicated with dark blue in part a), excluding genes also denoted as FLAGS.

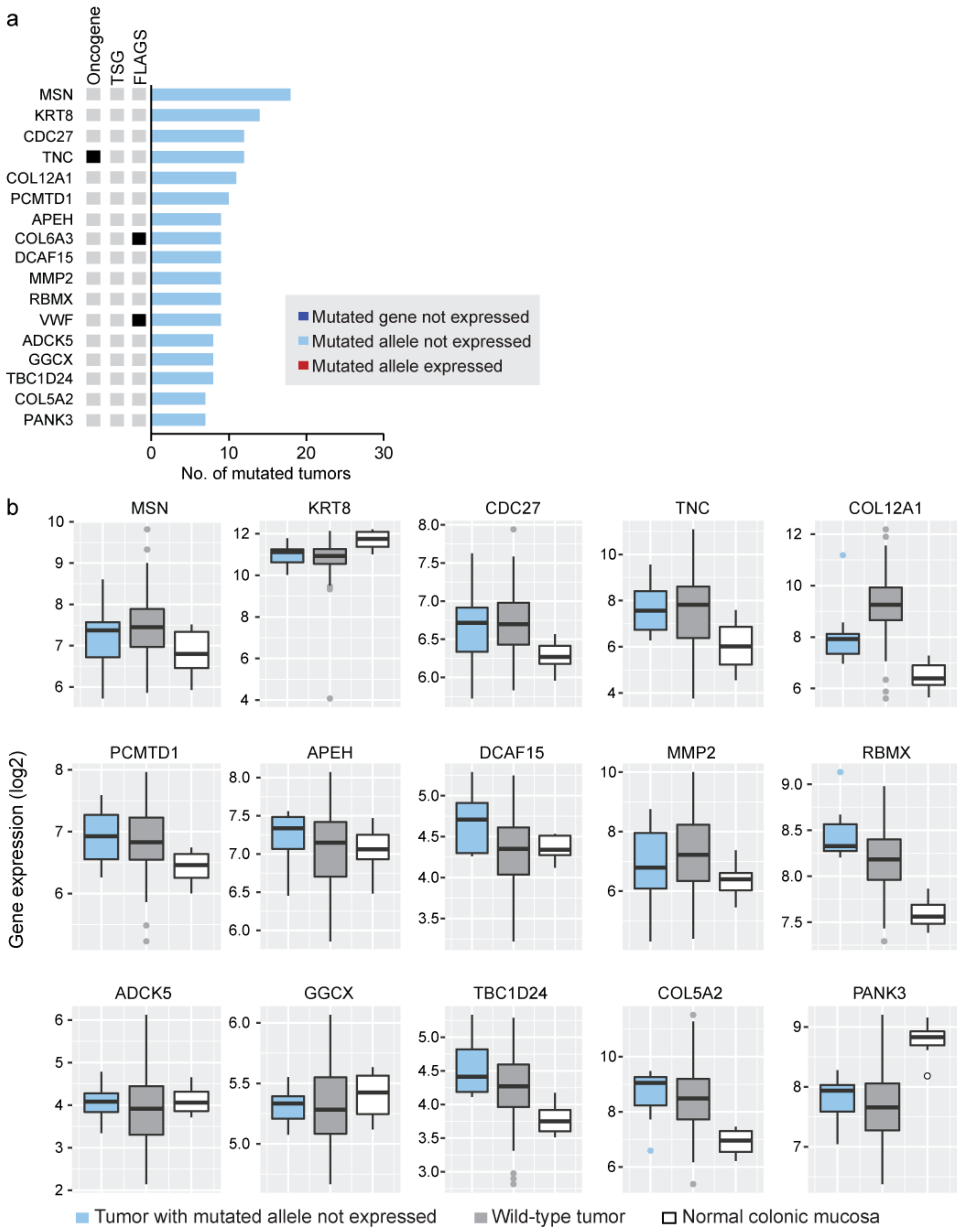

Figure S8. Genes with frequent non-expressed mutated alleles

a) Stacked bar plot of genes (n = 17) with non-expressed mutated alleles (light blue) in ≥7 tumors (excluding genes with expression of the mutated allele in a subset of tumors; plotted in Figure S6). Panels to the left indicate genes (black squares) that are denoted as oncogenes or tumor suppressor genes (TSG) in the CGC, and/or as FLAGS in Shyr et al., *BMC Med Genomics* 2014, **7**:64. Only mutated loci with sufficient coverage in the RNA sequencing data were included for analysis (Methods). **b)**

Boxplots of gene expression levels (log2-scale; normalized HTSeq-count data by the trimmed mean of M values and voom-transformation) across tumors (categorized according to mutation status for the respective gene) and normal colonic mucosa samples (*n* = 12). Genes denoted as FLAGS (*n* = 2) were not plotted.

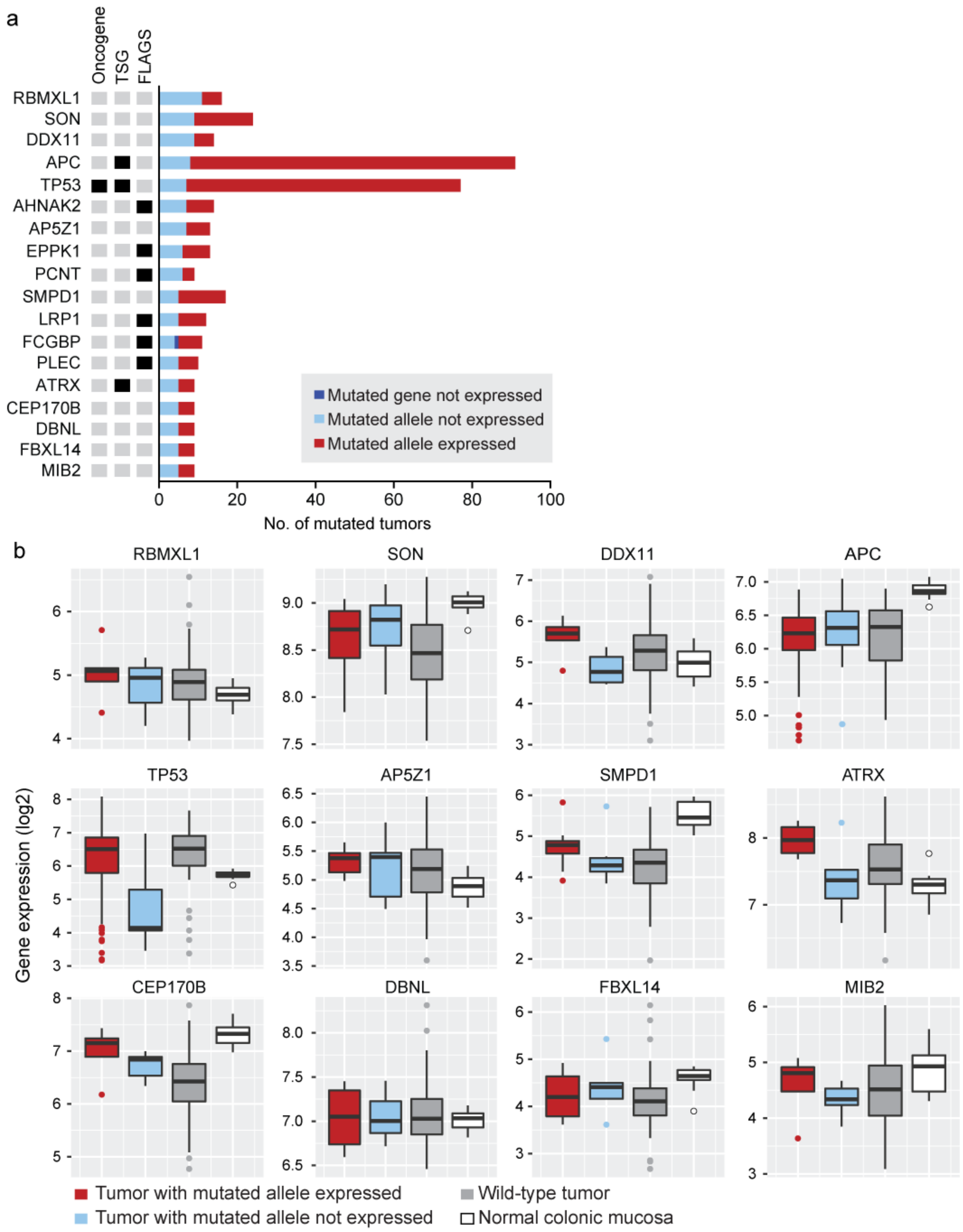

Figure S9. Genes with both expression and silencing of the mutated allele among tumors

a) Stacked bar plot of genes (*n* = 18) with expression of the mutated allele (red) in ≥3 tumors and wild-type only allelic expression (light blue) in ≥5 tumors. Panels to the left indicate genes (black squares) that are denoted as oncogenes or tumor suppressor genes (TSG) in the CGC, and/or as FLAGS in Shyr et al., *BMC Med Genomics* 2014, **7**:64. Only mutated loci with sufficient coverage in the RNA sequencing data were included for analysis (Methods). **b)** Boxplots of gene expression levels

(log2-scale; normalized HTSeq-count data by the trimmed mean of M values and voom-transformation) across tumors (categorized according to mutation status for the respective gene) and normal colonic mucosa samples (*n* = 12). Genes denoted as FLAGS (*n* = 6) were not plotted.

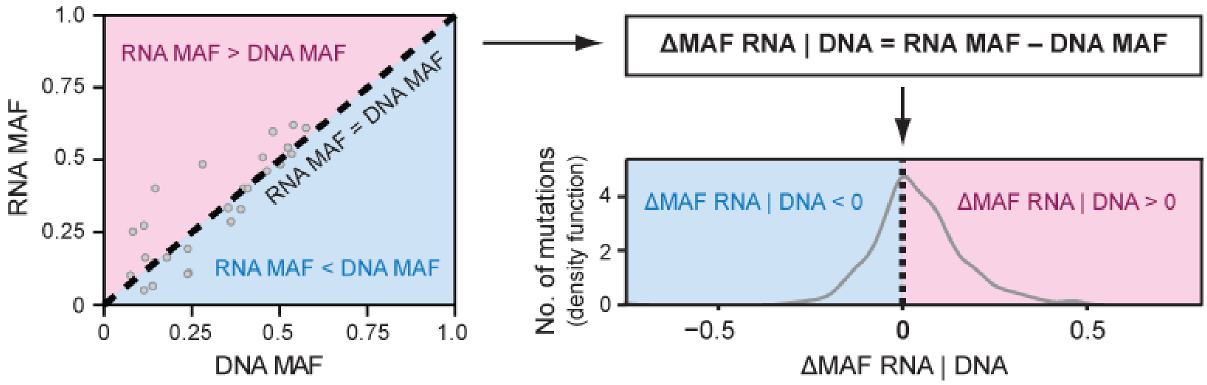

Figure S10. Illustration of estimates used to describe relative allelic expression of mutations. Allele-specific mutation expression was estimated based on the MAF in the RNA sequencing data, relative to the MAF on the DNA level (exome sequencing data). Scatter plot to the left represents the RNA MAF *versus* DNA MAF for individual mutations (grey points). Density plot on the bottom right summarizes the difference between the RNA MAF and DNA MAF (mutation-wise) for a set of mutations. Dashed black line indicates equal expression level of the mutated and wild-type alleles at a mutated locus, adjusted for the MAF on the DNA level ([RNA MAF = DNA MAF], also denoted [ΔMAF RNA|DNA = 0]). Pink background color indicates preferential expression of the mutated compared to the wild-type allele ([RNA MAF > DNA MAF], or [ΔMAF RNA|DNA > 0]). Blue background color indicates lower relative expression of the mutated compared to the wild type allele ([RNA MAF < DNA MAF], or [ΔMAF RNA|DNA < 0]).

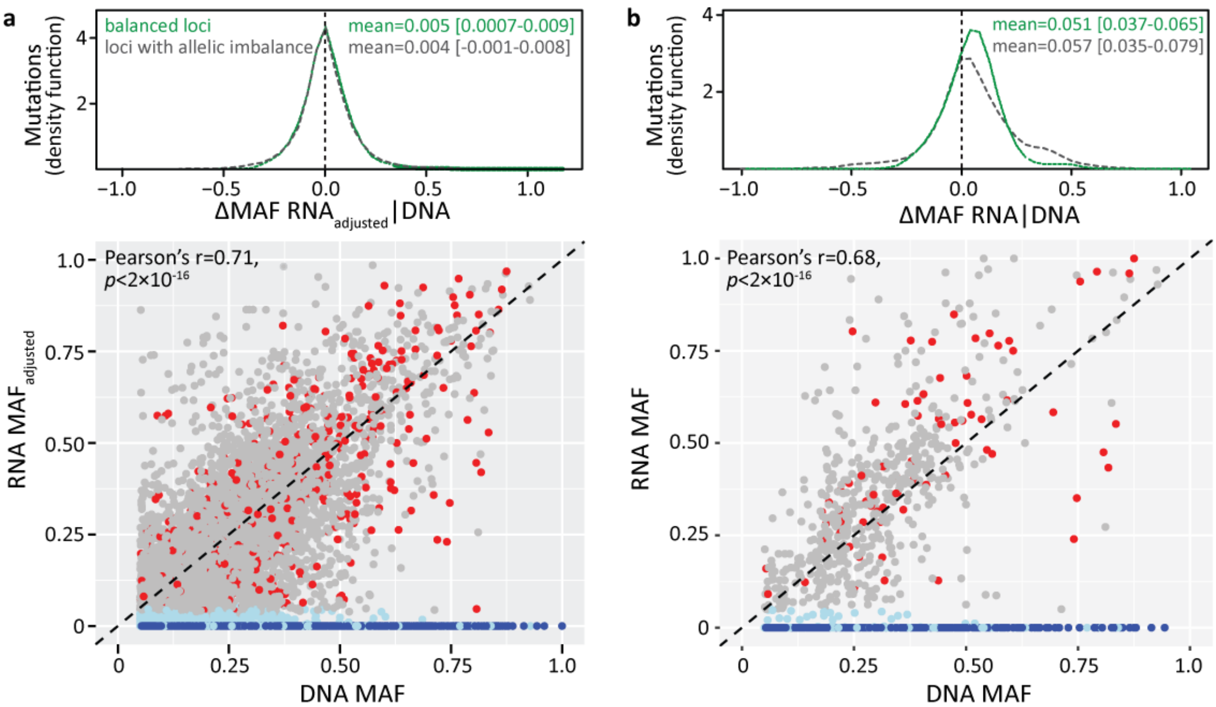

DNA MAF vs. RNA MAF$_{adjusted}$	Pearson's r	*p*-value
balanced loci	0.60	$<2\times10^{-16}$
unbalanced loci	0.74	$<2\times10^{-16}$
all loci, adjusted by tumor purity	0.66	$<2\times10^{-16}$

DNA MAF vs. RNA MAF	Pearson's r	*p*-value
balanced loci	0.61	$<2\times10^{-16}$
unbalanced loci	0.67	$<2\times10^{-16}$
all loci, adjusted by tumor purity	0.63	$<2\times10^{-16}$

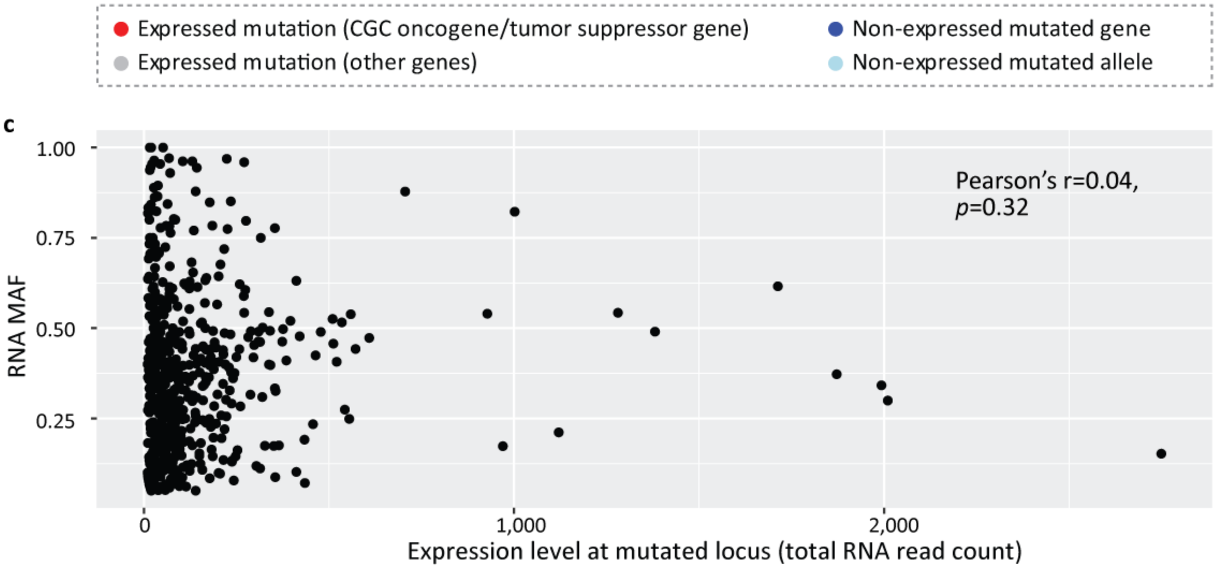

Figure S11. RNA and DNA level MAFs in primary MSS CRCs in the in-house series

a) Includes all mutations in all samples (with adjustments for RNA MAFs) and **b)** includes all mutations in 11 samples from 6 tumors with combined DNA/RNA extraction (no RNA MAF adjustment needed). The upper panels show the difference between the RNA-level and DNA-level MAFs of expressed mutations (ΔMAF RNA|DNA), plotted as the distribution (density function) of the mutations at DNA copy number balanced and unbalanced loci separately. ΔMAF RNA|DNA above or below 0 indicate mutated loci with preferential expression of the mutated or wild-type alleles, respectively. The middle panels show scatter plots of RNA-level *versus* DNA-level MAFs, indicating that the majority of mutations have expression levels as expected according to the allelic DNA frequency (clustering close to the dashed line, which represents ΔMAF RNA|DNA = 0). However, a

slight skewedness towards higher relative expression of mutated alleles was indicated in the analyses where RNA MAF adjustment was not needed. Correlation analyses were performed for expressed mutations only, and the tables in the lower panels show results for DNA copy number balanced and unbalanced loci separately, as well as after adjustment of RNA-level and DNA-level MAFs by the estimated tumor purity of the samples . **c)** The RNA-level MAFs in the 11 samples were no adjustments were needed were not associated with the total expression level at the mutated loci.

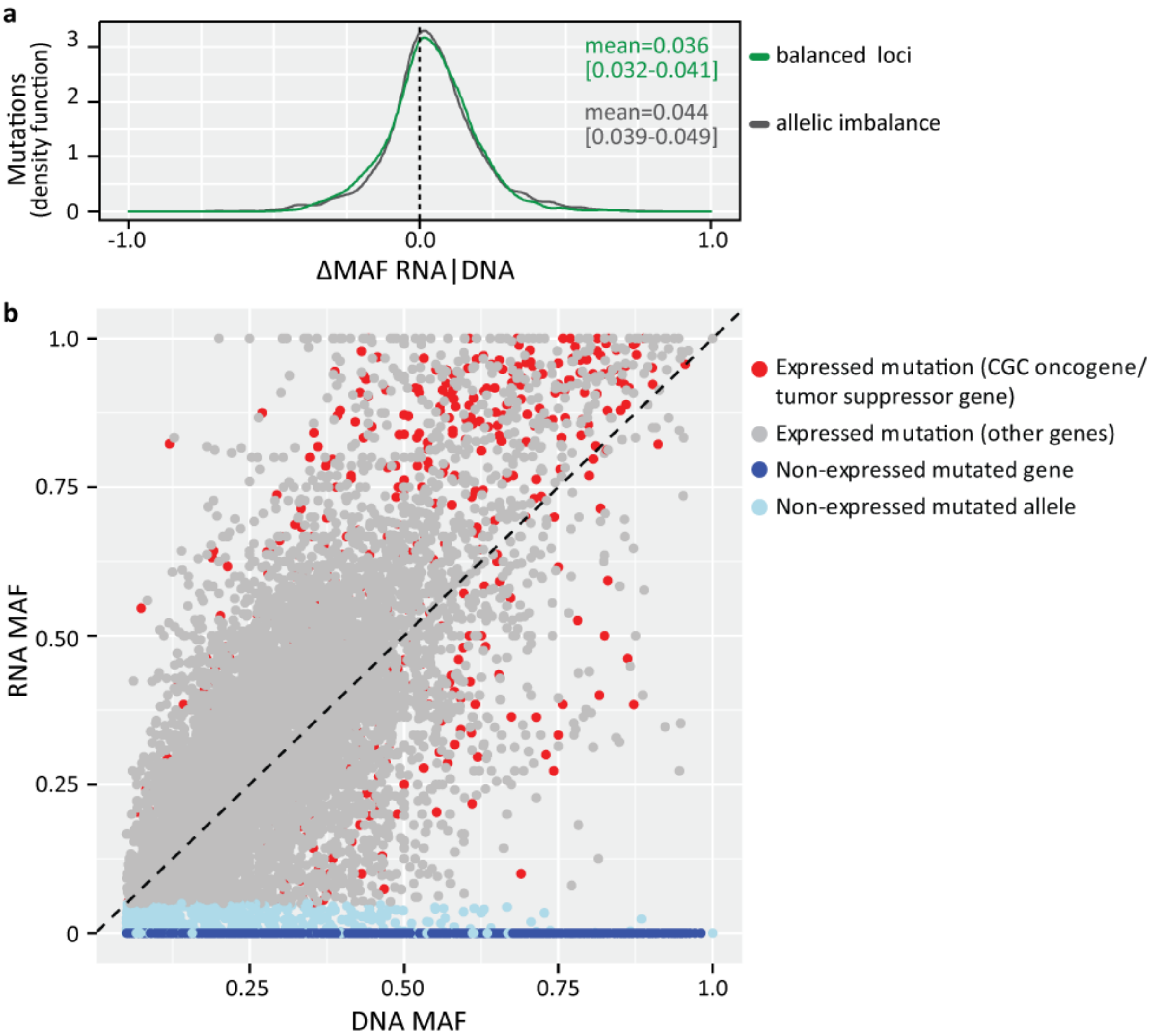

**DNA MAF vs. RNA MAF** (expressed mut)	**Pearson's r**	***p*-value**
overall (all expressed mutations)	0.69	$<2\times10^{-16}$
oncogenes/tumor suppressor genes	0.74	$<2\times10^{-16}$
other genes	0.67	$<2\times10^{-16}$
balanced loci	0.46	$<2\times10^{-16}$
allelic imbalance	0.76	$<2\times10^{-16}$

Figure S12. RNA MAF *versus* DNA MAF in TCGA

Validation analyses in 241 MSS colon cancers from TCGA supported **a)** a slight overexpression of mutated compared to wild-type alleles independent of allelic imbalance, although **b)** there was strong correlation between DNA level and RNA level MAFs of expressed mutations. The density plot in a) shows the difference between the RNA-level and DNA-level MAFs of expressed mutations (ΔMAF RNA|DNA). ΔMAF RNA|DNA above or below 0 indicate mutated loci with preferential expression of the mutated or wild-type alleles, respectively. Only expressed mutations were included for statistical correlation analyses in b).

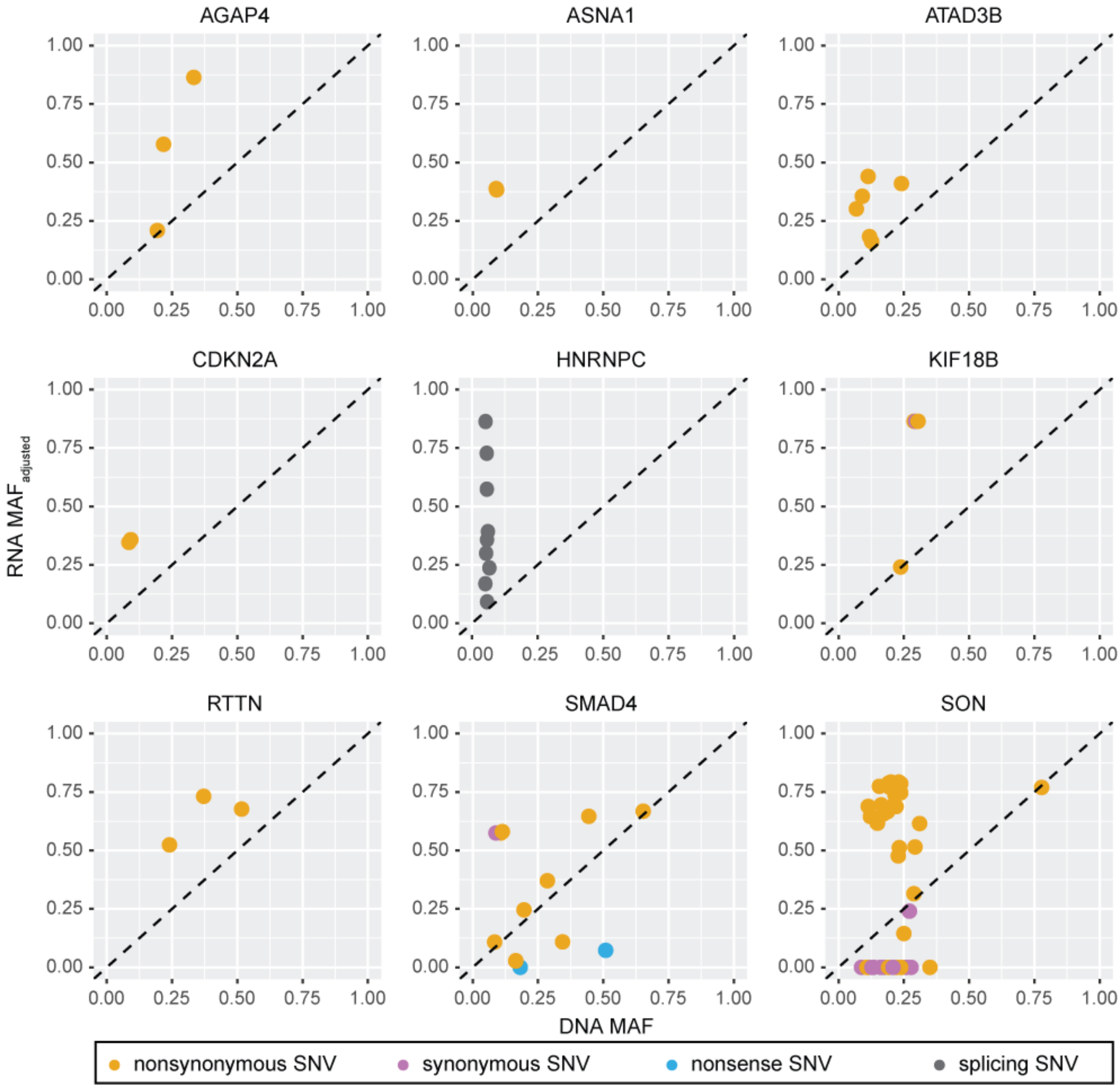

Figure S13. Genes with high relative allelic expression of mutations

Scatter plots of the mutant allele fraction on the RNA (vertical axes) *versus* DNA level (horizontal axes) for genes (n = 9; excluding TP53 which is plotted in Figure 4a) with high relative allelic mutation expression (ΔMAF $RNA_{adjusted}|DNA > 0.25$) in at least two tumors across the in-house series. Mutations are colored according to mutation type as indicated.

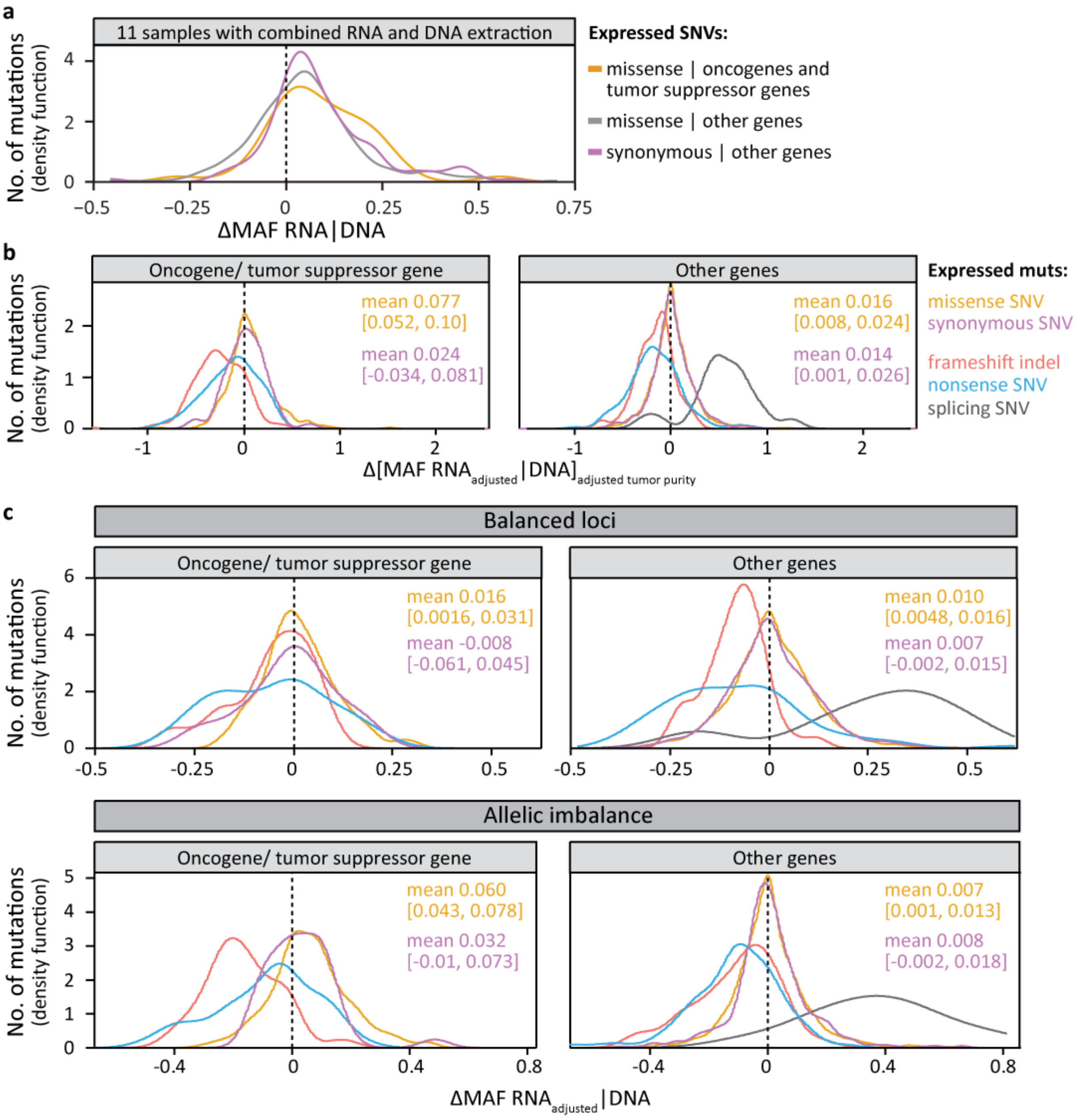

Figure S14. RNA MAF *versus* DNA MAF in in-house series according to mutation/target gene category

a) Analyses only of the 11 samples with combined RNA and DNA extraction supported a higher relative expression of mutated alleles specifically at missense SNV loci in oncogenes and tumor suppressor genes, indicating that the results were not biased by RNA MAF adjustments or intra-tumor heterogeneity. **b)** Adjustment of RNA-level and DNA-level MAFs by the estimated tumor purity of the samples (based on DNA copy number profiles, estimated by FACETS) did not have a large impact on the relative allelic expression patterns. **c)** Comparisons of relative allelic expression patterns at copy number balanced and unbalanced loci indicated that the higher expression levels of missense SNVs (compared to synonymous SNVs) specifically in oncogenes and tumor suppressor genes were driven by allelic imbalance. All plots show the difference between the RNA-level and DNA-level MAFs of expressed mutations (ΔMAF RNA|DNA) plotted as the distribution (density function) within categories of target genes and mutation types (color-coded). ΔMAF RNA|DNA above

or below 0 indicate mutated loci with preferential expression of the mutated or wild-type alleles, respectively. The plots only include mutation groups with >5 mutations. The mean and 95% CIs of the ΔMAF RNA|DNA of missense SNVs and synonymous SNVs are included in the plots according to the color code.

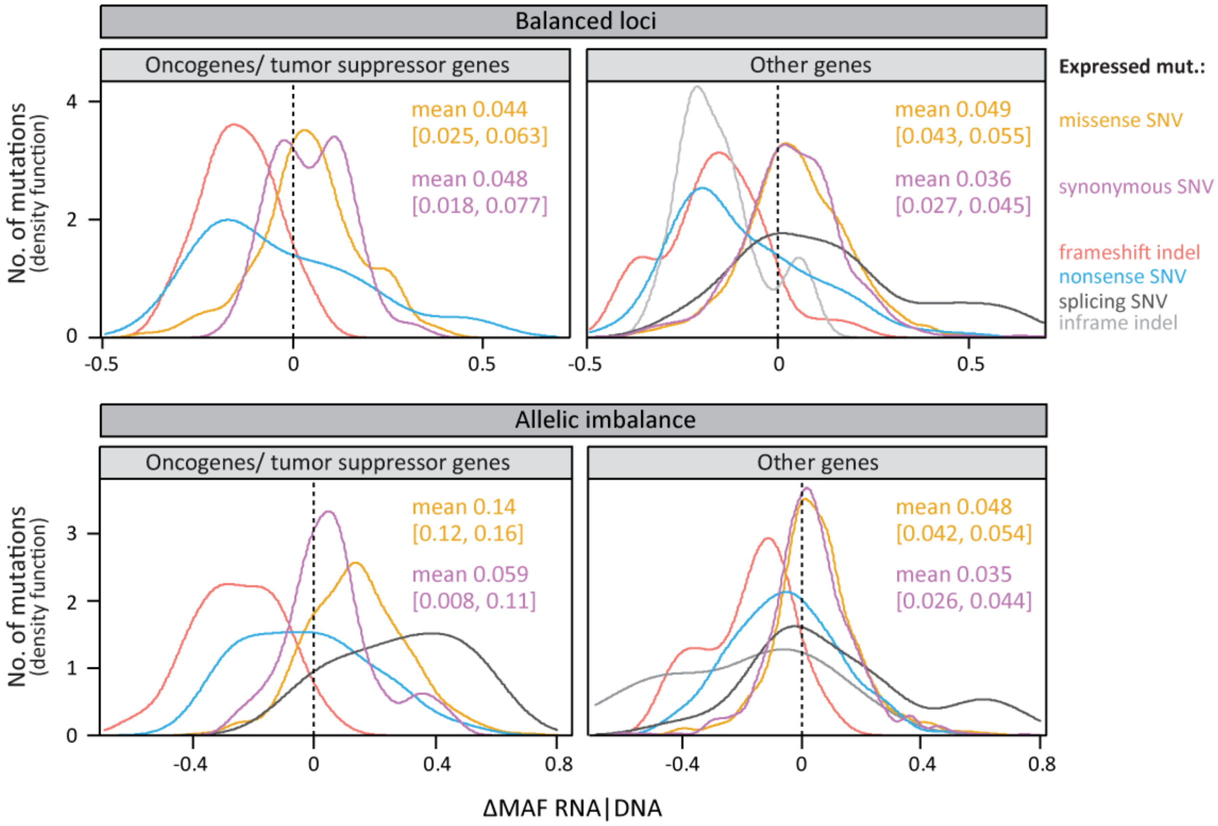

Figure S15. Relative allelic mutation expression in TCGA data according to allelic copy number balance

Validation analyses among MSS colon cancers from TCGA (corresponding plot to Figure S14c for the in-house data) supported that the higher expression level of missense SNVs (compared to synonymous SNVs) specifically in oncogenes and tumor suppressor genes was driven by allelic imbalance at the mutated loci. The difference in the mean ΔMAF RNA|DNA between missense SNVs and synonymous SNVs was statistically significant only for oncogenes/tumor suppressor genes and only at loci with allelic imbalance, as indicated by the 95% CI of the mean.

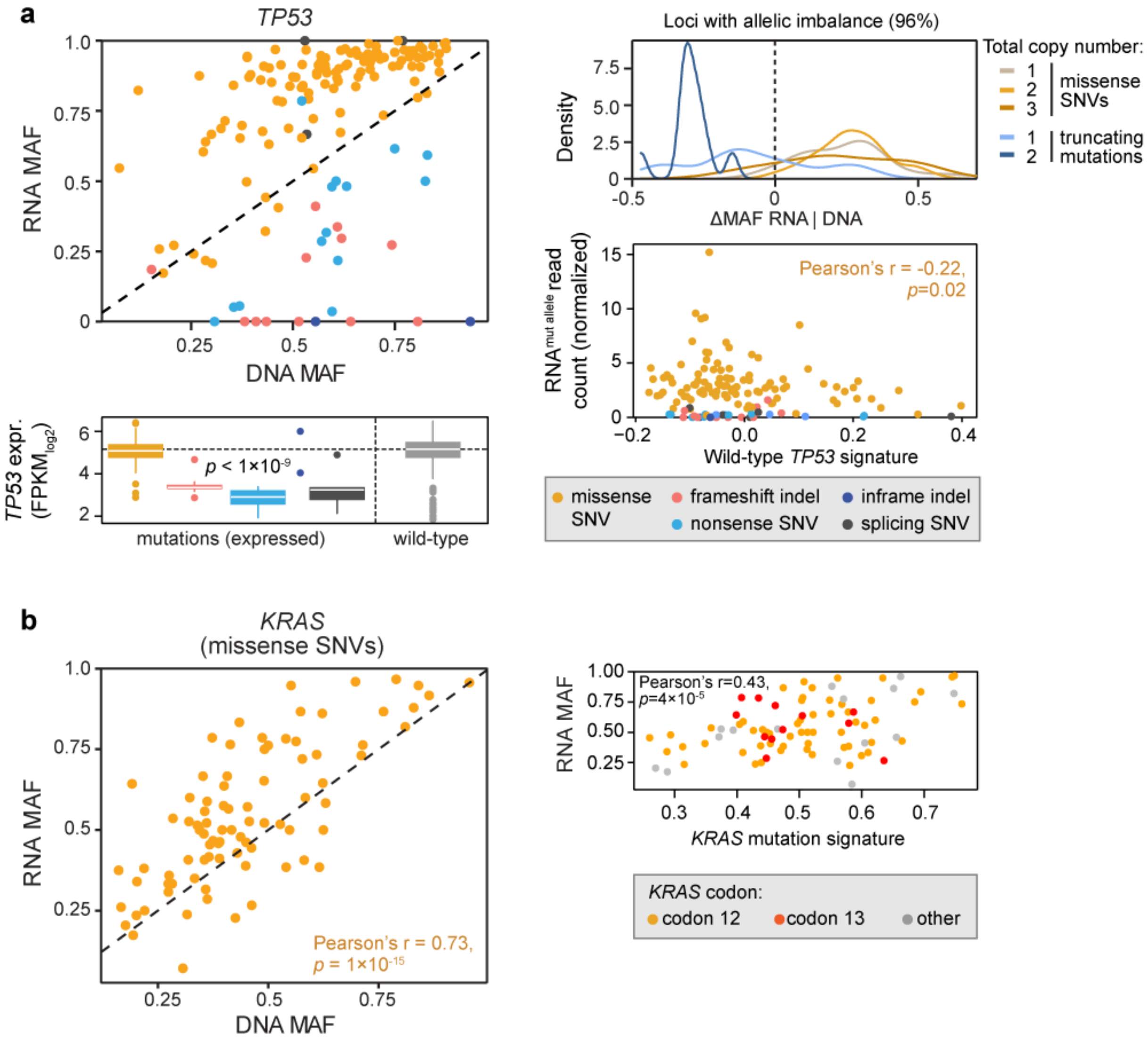

Figure S16. Allele-specific expression of *TP53* and *KRAS* mutations among 241 primary colon cancers in the TCGA validation series

a) Scatter plot (top left) of RNA-level *versus* DNA-level MAFs in *TP53* shows skewedness of mutant allele-specific expression levels according to mutation types (color-coded as indicated) and DNA copy numbers at the mutated loci (summarized in the density plot in the top right panel). The higher mutant allele-specific expression level of missense SNVs than putative truncating mutations (nonsense SNVs and frameshift indels) corresponded with the gene expression level of *TP53* (right bottom panel). The allele-specific read counts of missense SNVs were negatively correlation with a gene expression signature of wild-type *TP53*, while the expression levels of truncating mutations were too low for accurate analyses (right bottom panel). **b)** *KRAS* was only targeted by missense SNVs, and these mutations had largely proportional RNA- and DNA-level MAFs. The RNA MAFs were significantly correlated with a sample-wise gene expression signature of mutant *KRAS*.

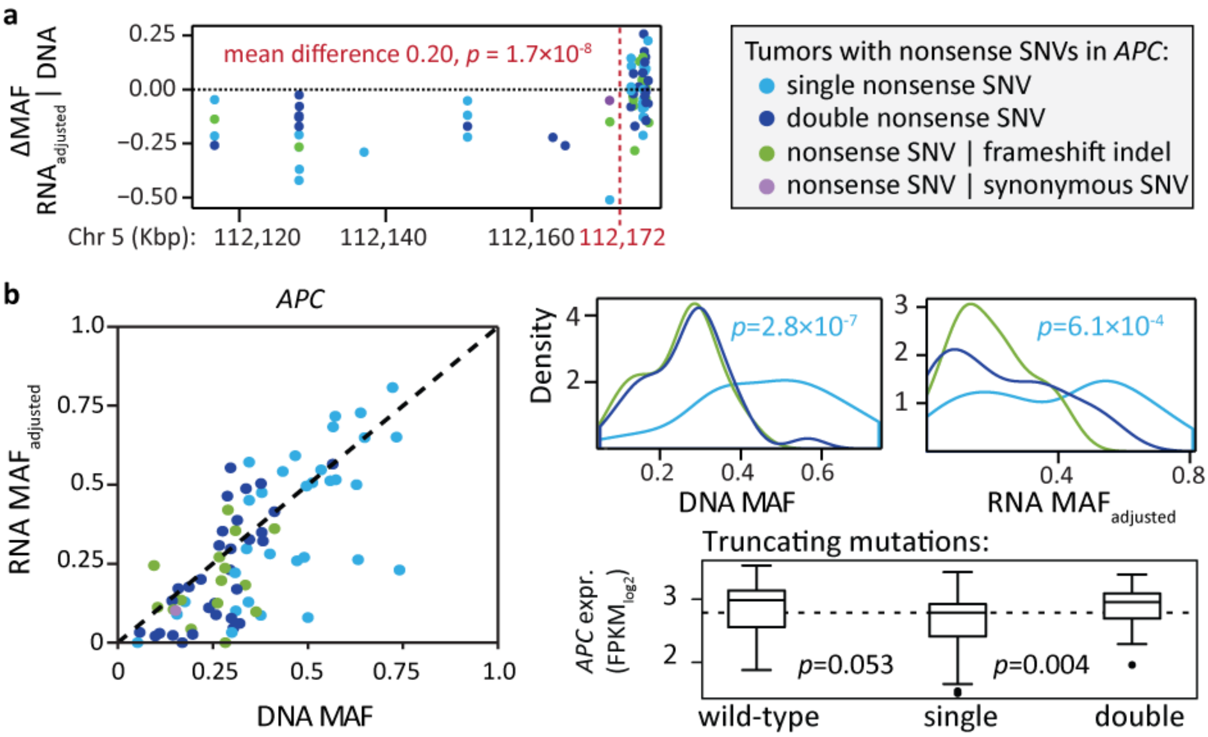

Figure S17. Allele-specific expression of *APC* mutations in the in-house series of primary CRCs
a) The difference between the RNA-level and DNA-level MAFs of nonsense SNV in *APC* (ΔMAF $RNA_{adjusted}$ | DNA) varied according to the mutated locus, and showed significantly higher allele-specific expression levels in the 3' region of the gene, compared to more upstream mutations. **b)** Scatter plot (left) and distribution plots (density functions; upper right) of RNA and DNA MAFs of nonsense SNVs in *APC*, colored according to co-occurrence with other *APC* mutations in the same tumor. Tumors with single nonsense mutations in *APC* (light blue) had significantly higher DNA and RNA MAFs than tumors with two mutations. Double putative truncating mutations (nonsense SNVs and frameshift indels) were associated with a similar *APC* gene expression level to tumors with wild-type *APC*, and both had higher *APC* expression than single mutated tumors (p = 0.004 and 0.053, respectively, by Welch's t-test), but with low differential expression (mean difference 0.25 and 0.20, respectively, on a log2-scale).

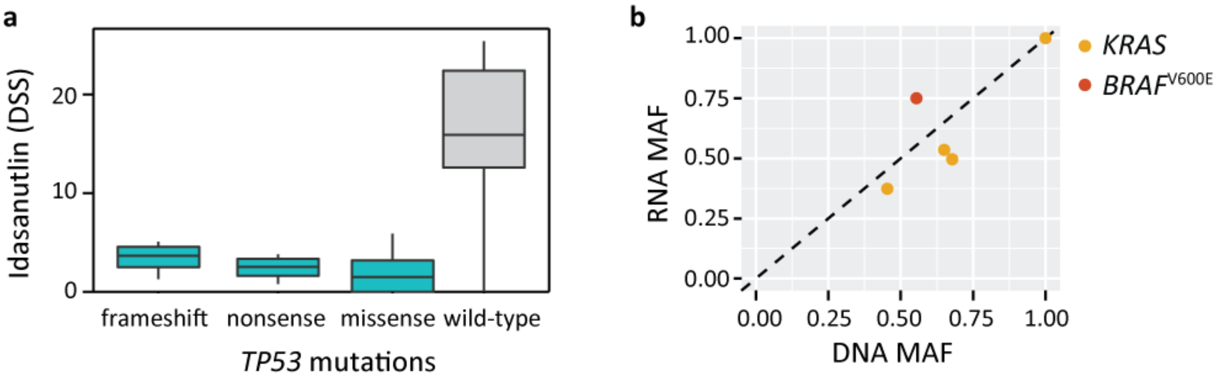

Figure S18. Allele-specific mutation expression and drug sensitivity in CRC cell lines

a) In the panel of 29 unique CRC cell lines, sensitivity to the MDM2/TP53-inhibitor idasanutlin (plotted as drug sensitivity scores, DSS) was significantly higher among *TP53* wild-type compared with mutated samples, but there was no difference in sensitivity between the different *TP53* mutation types. **b)** Among the 7 mutated cell lines selected for RNA sequencing, the RNA-level and DNA-level mutant allele fractions (MAFs) of *RAS*/*BRAF* mutations were proportional. DNA-level MAFs were from “kinome” sequencing of the cell lines (Berg et al., Mol Cancer 2017;16:116). The MAFs of *TP53* mutations were 1 at both the DNA-level and RNA-level in all cell lines and therefore not plotted.

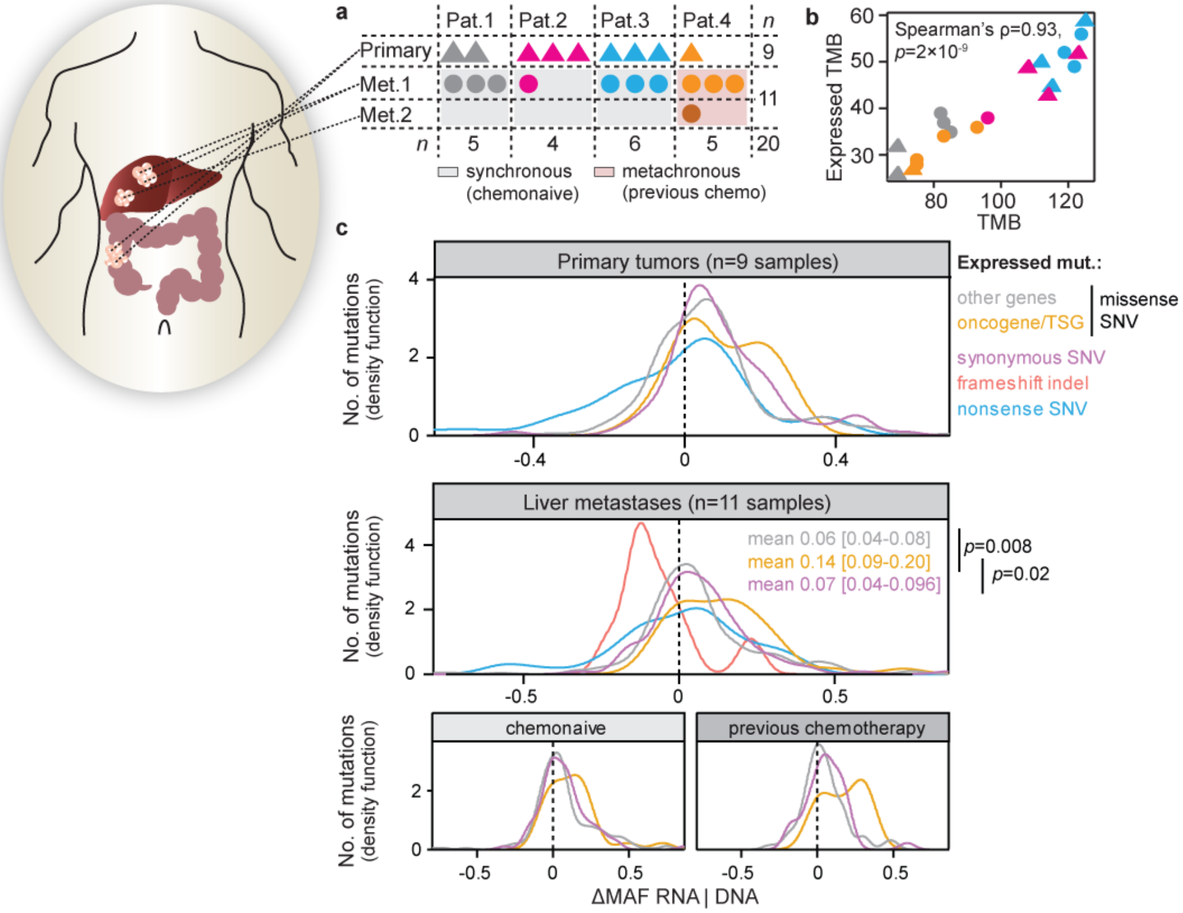

Figure S19. Minimal evolutionary changes in mutation expression during metastasis
a) Potential intra-patient heterogeneity in mutation expression was analyzed in totally 20 samples from the primary tumor and matched liver metastases from 4 patients. One patient (patient 4) had metachronous metastases and was treated with chemotherapy prior to sampling of the metastases (in the adjuvant setting for the stage II primary tumor). **b)** The expressed tumor mutational burden (TMB) was correlated with the TMB across the primary-metastasis sample set, with a range of patient-wise Spearman's correlations of 0.56-0.97. **c)** Patterns of allelic mutation expression according to mutation type and target gene category were similar among metastases and primary tumors, including a higher ΔMAF RNA|DNA of missense SNVs in oncogenes and tumor suppressor genes (TSG) compared both to missense SNVs in other genes and to synonymous SNVs. Previous exposure to chemotherapy did not impact this result. Figures S20-S23 below show strong proportionality in the DNA MAF, RNA MAF, ΔMAF RNA|DNA, and RNA read counts (analyzed separately) of the mutated allele among samples in patient-wise comparisons, consistent with uniform tumor heterogeneity and the "big bang" model of CRC growth.

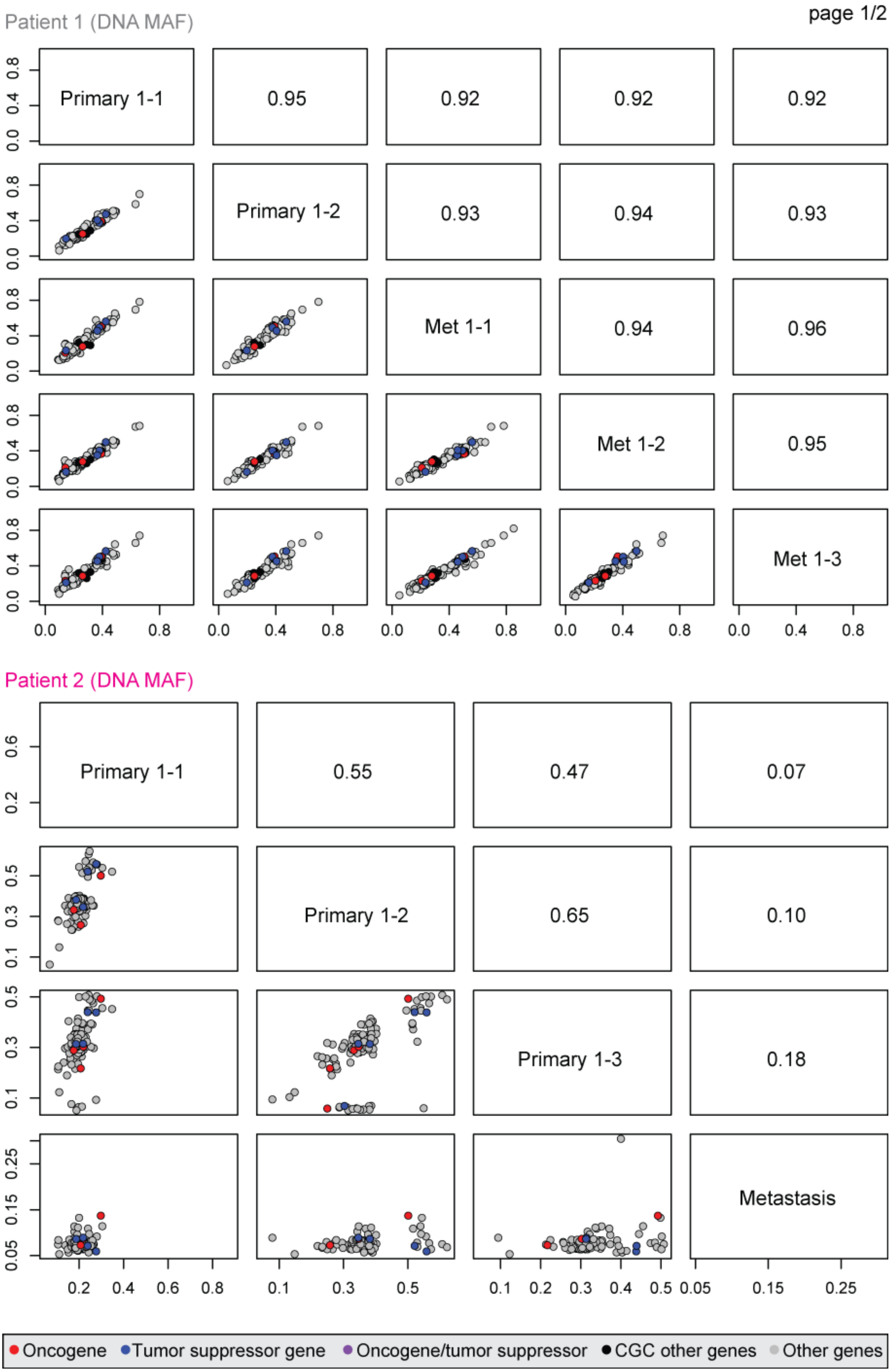
Patient 1 (DNA MAF)
page 1/2
Primary 1-1
0.95
0.92
0.92
0.92
Primary 1-2
0.93
0.94
0.93
Met 1-1
0.94
0.96
Met 1-2
0.95
Met 1-3
0.0
0.4
0.8
Patient 2 (DNA MAF)
Primary 1-1
0.55
0.47
0.07
Primary 1-2
0.65
0.10
Primary 1-3
0.18
Metastasis
0.2
0.6
0.1
0.3
0.5
0.05
0.15
0.25
0.4
0.8
Oncogene
Tumor suppressor gene
Oncogene/tumor suppressor
CGC other genes
Other genes

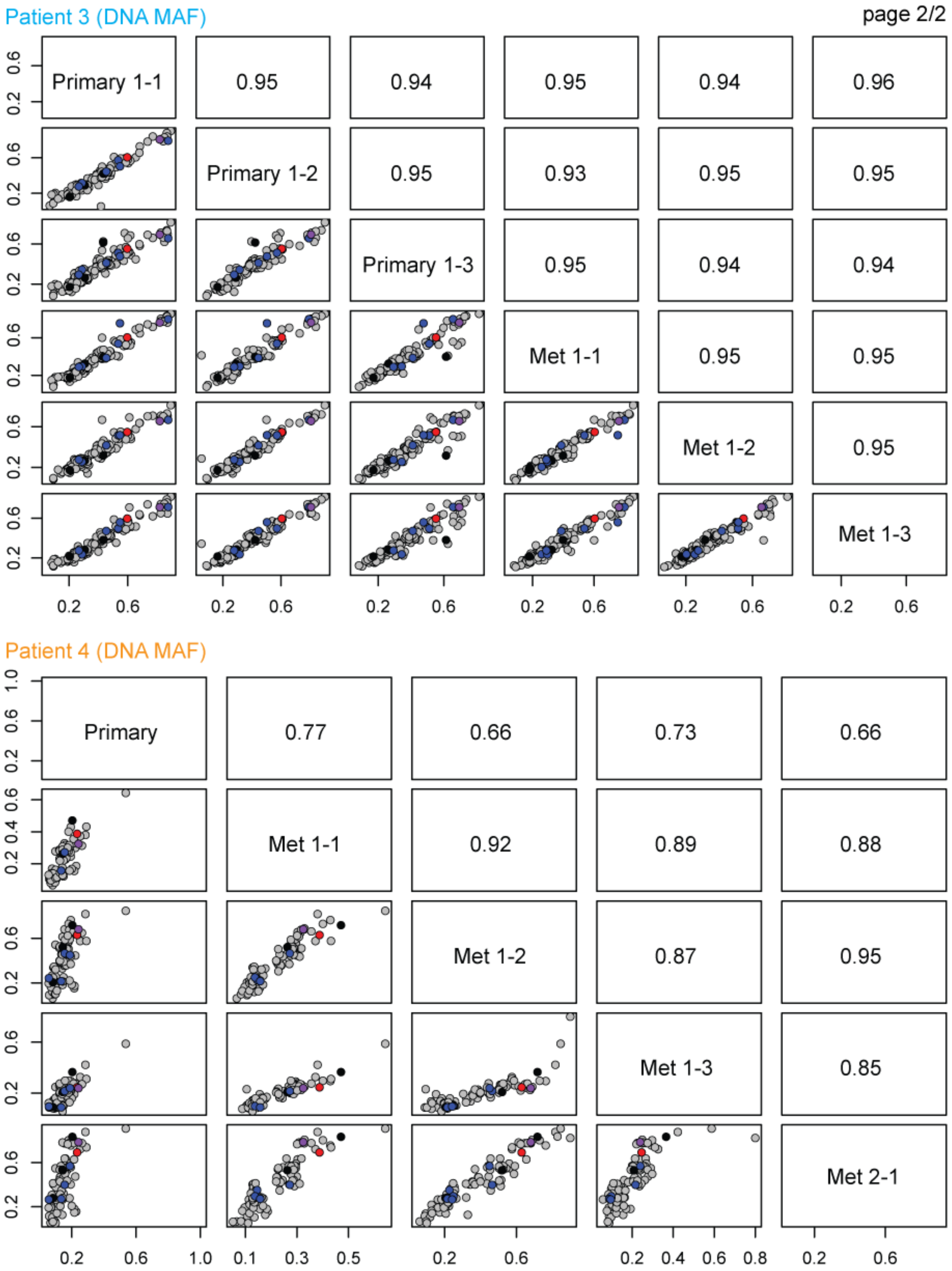

Figure S20. Comparisons of DNA MAFs among multiple tumor samples from each of 4 patients
Each scatter plot matrix represents pair-wise comparisons of tumor samples from each of 4 patients, with DNA MAFs plotted on both the vertical and horizontal axes (bottom left panels), and with the corresponding Spearman's ρ indicated in the upper right panels. Each dot represents a mutation colored according to target gene category (all mutations found in at least one sample per patient were included). The large range of DNA MAFs indicated that the tumors were subclonal, emphasized

by the separation of mutations into clusters according to MAFs, in particular in patients 2 and 4. The strong proportionality in DNA MAFs between sample pairs indicated that the tumor heterogeneity was uniform, both in intra-tumor comparisons (primary and metastases), in primary-metastasis comparisons, and in inter-metastatic comparisons (the latter was analyzed in patient 4 only). The only exception from this proportionality was found between samples 2 and 3 from the primary tumor of patient 2 (Primary 1-2 and 1-3), indicating spatial intra-tumor heterogeneity. In patient 2, sample 1 from the primary tumor and the metastatic sample had low cancer cell fractions (low DNA MAFs), likely contributing to the weaker Spearman's ρ observed between samples in this patient.

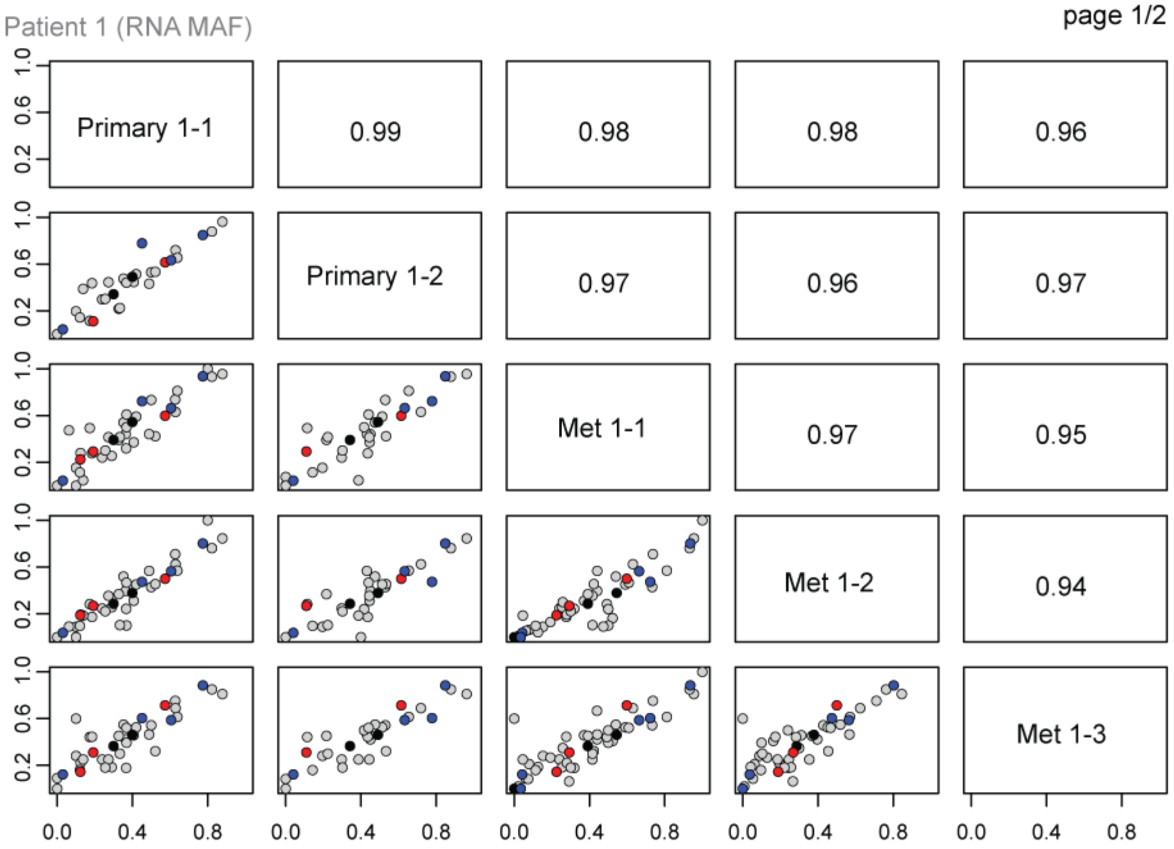
Patient 1 (RNA MAF)
Primary 1-1
0.99
0.98
0.98
0.96
Primary 1-2
0.97
0.96
0.97
Met 1-1
0.97
0.95
Met 1-2
0.94
Met 1-3
0.0 0.4 0.8
0.2 0.6 1.0

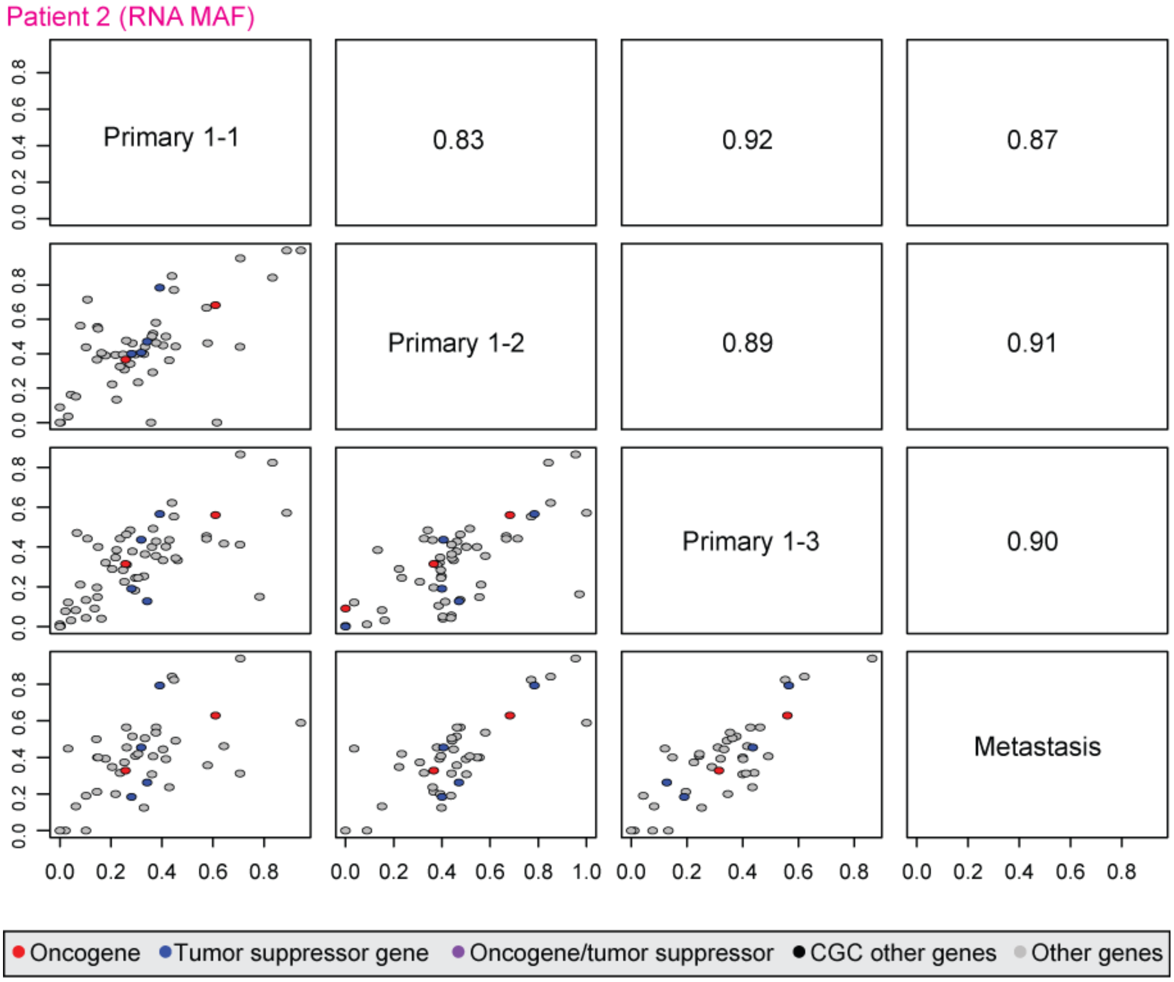
Patient 2 (RNA MAF)
Primary 1-1
0.83
0.92
0.87
Primary 1-2
0.89
0.91
Primary 1-3
0.90
Metastasis
0.0 0.2 0.4 0.6 0.8 1.0
Oncogene
Tumor suppressor gene
Oncogene/tumor suppressor
CGC other genes
Other genes

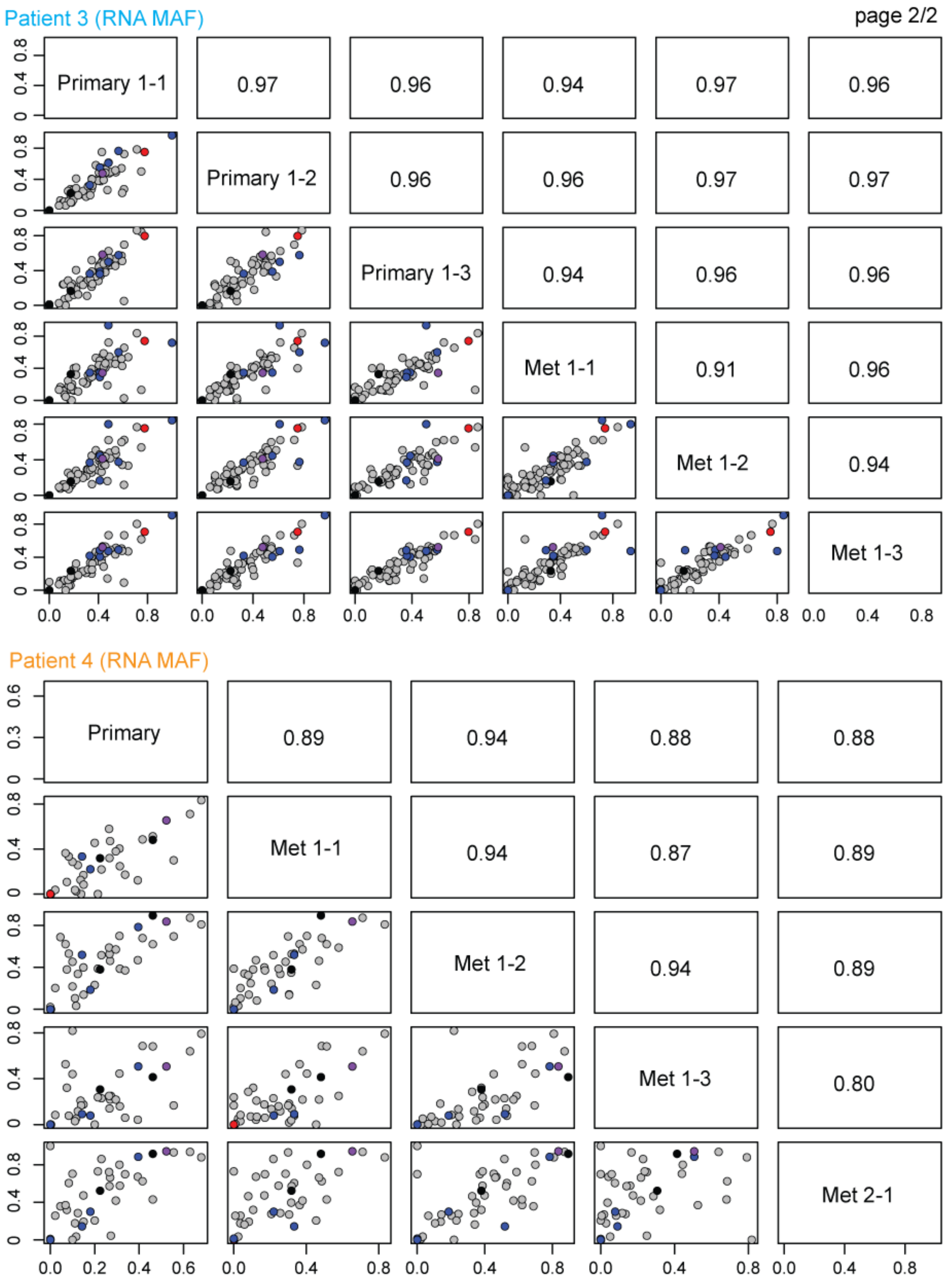

Figure S21. Comparisons of RNA MAFs among multiple tumor samples from each of 4 patients
Each scatter plot matrix represents pair-wise comparisons of tumor samples from each of 4 patients, with RNA MAFs plotted on both the vertical and horizontal axes (bottom left panels), and with the corresponding Spearman's ρ indicated in the upper right panels. Each dot represents a mutation, colored according to target gene category (only mutations found in expressed genes are plotted).

This shows that the proportionality of mutations between sample pairs found on the DNA level was recapitulated on the RNA level. The patient-wise median Spearman's ρ of RNA MAFs was 0.97 (95% CI 0.95-0.98; patient 1), 0.90 (95% CI 0.83-0.92; patient 2), 0.96 (95% CI 0.94-0.97; patient 3), and 0.89 (95% CI 0.87-0.94; patient 4).

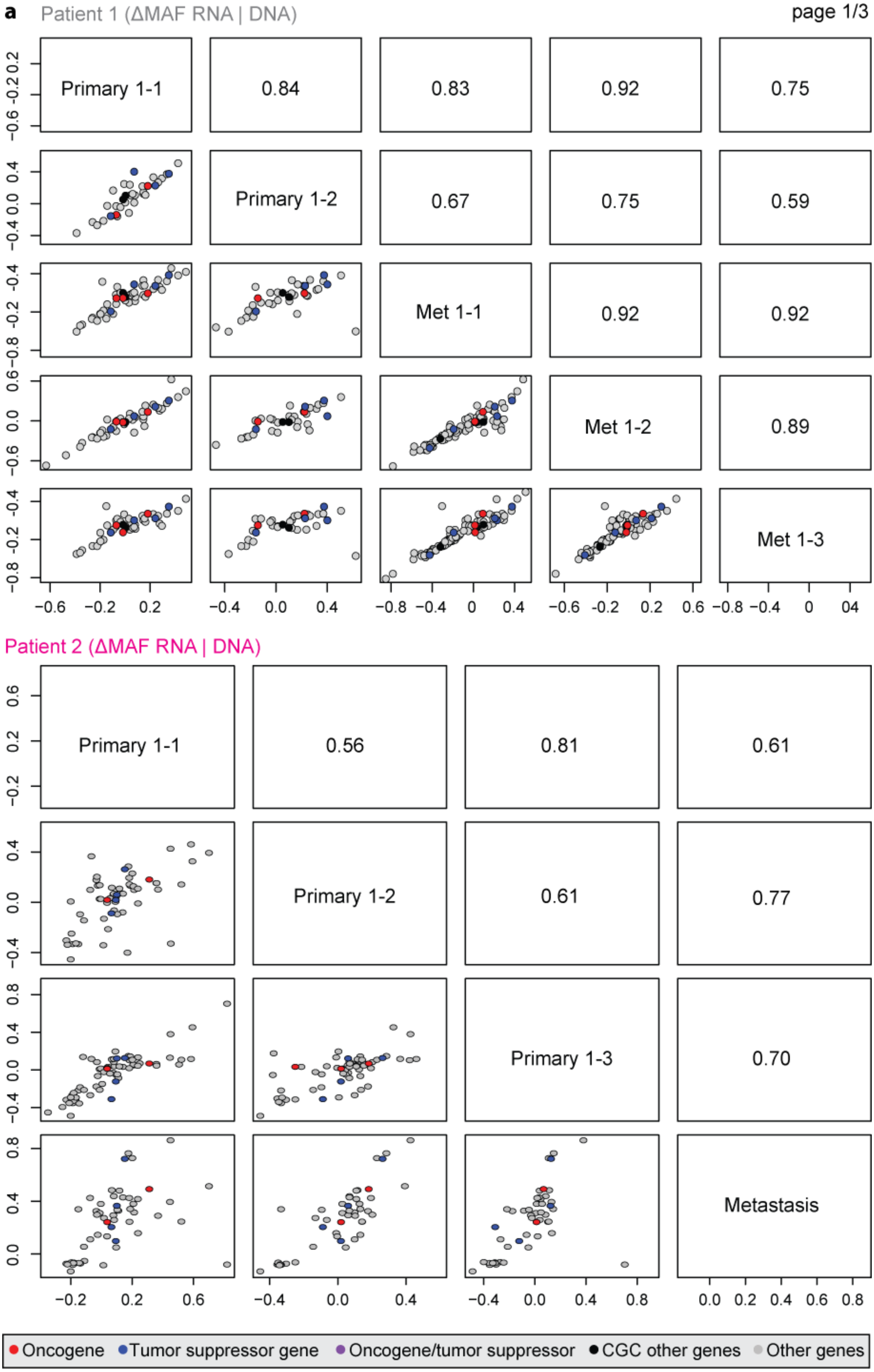
a
Patient 1 (ΔMAF RNA | DNA)
page 1/3
Primary 1-1
0.84
0.83
0.92
0.75
Primary 1-2
0.67
0.75
0.59
Met 1-1
0.92
0.92
Met 1-2
0.89
Met 1-3
Patient 2 (ΔMAF RNA | DNA)
Primary 1-1
0.56
0.81
0.61
Primary 1-2
0.61
0.77
Primary 1-3
0.70
Metastasis
Oncogene
Tumor suppressor gene
Oncogene/tumor suppressor
CGC other genes
Other genes

Patient 3 (ΔMAF RNA | DNA)

page 2/3

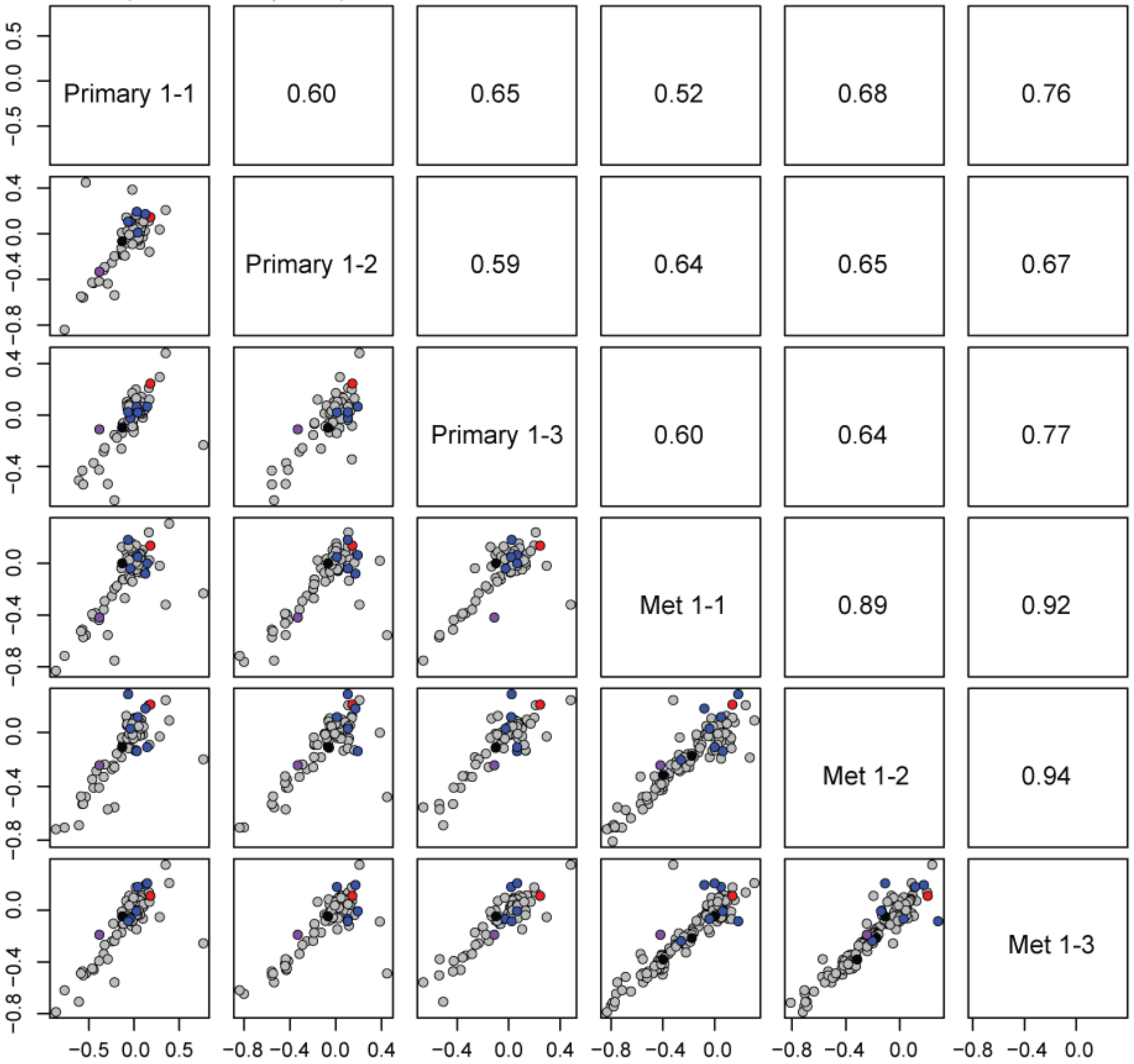

Patient 4 (ΔMAF RNA | DNA)

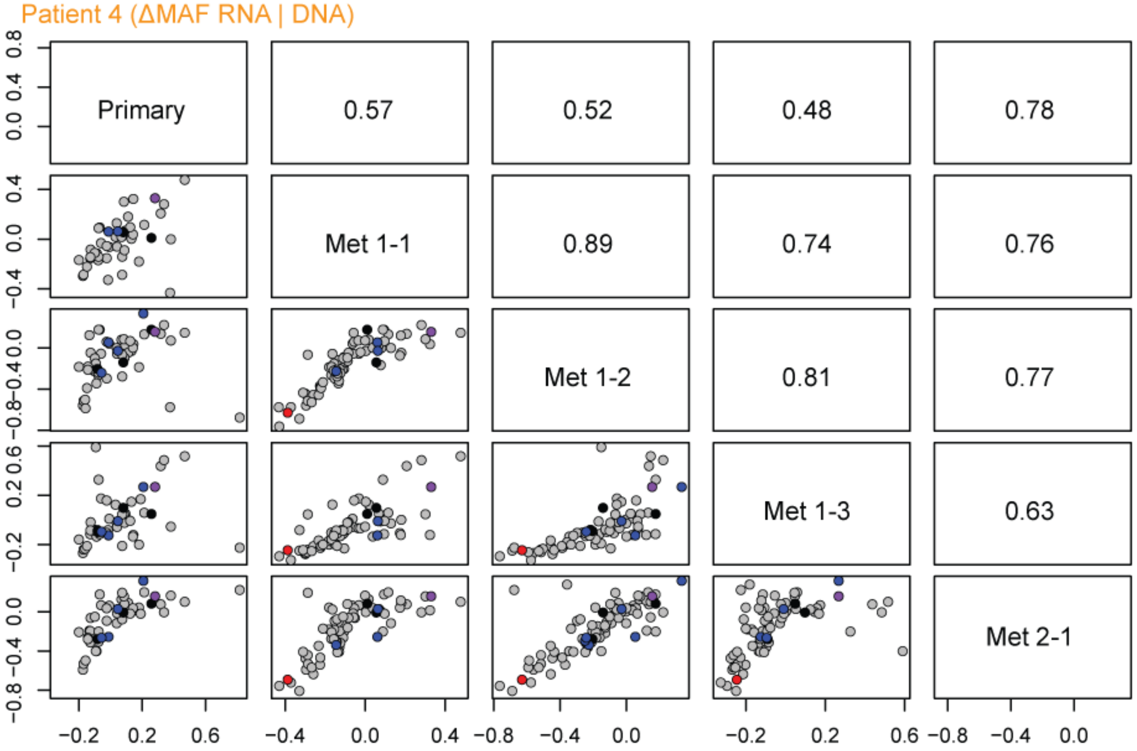

b

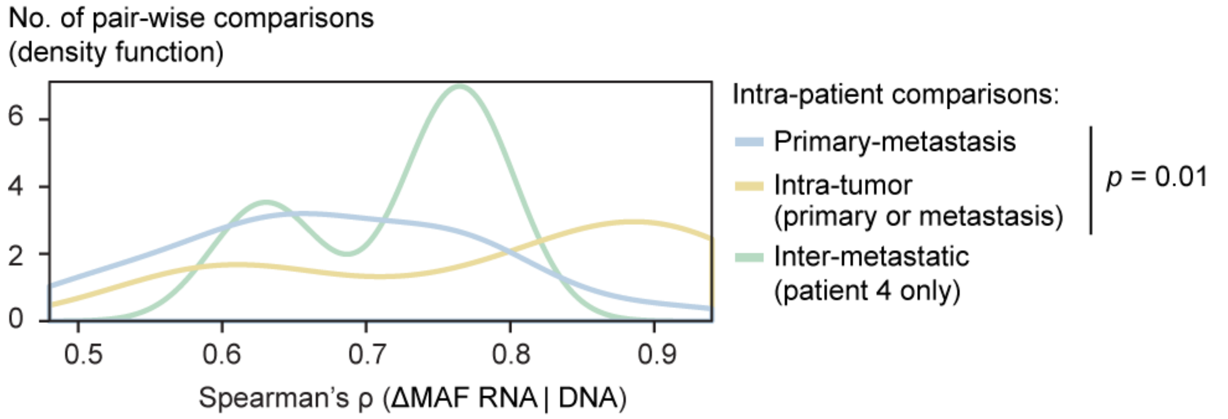

Figure S22. Comparisons of ΔMAF RNA|DNA among multiple tumor samples from each of 4 patients

a) Each scatter plot matrix represents pair-wise comparisons of tumor samples from each of 4 patients, with ΔMAF RNA|DNA plotted on both the vertical and horizontal axes (bottom left panels), and with the corresponding Spearman's ρ indicated in the upper right panels. Each dot represents a mutation, colored according to target gene category (only mutations found in expressed genes are plotted). **b)** Density plot of Spearman's correlation coefficients (ρ) of ΔMAF RNA|DNA, summarizing all sample comparisons and colored according to comparison type. Primary-metastasis comparisons had somewhat weaker correlation coefficients than intra-tumor comparisons (p = 0.01 by Welch's t-test) and inter-metastatic comparisons (only 3 comparisons between two metastatic deposits from patient 4).

page 1/2

Patient 1 ($RNA^{mut\ allele}$ read count [normalized]):

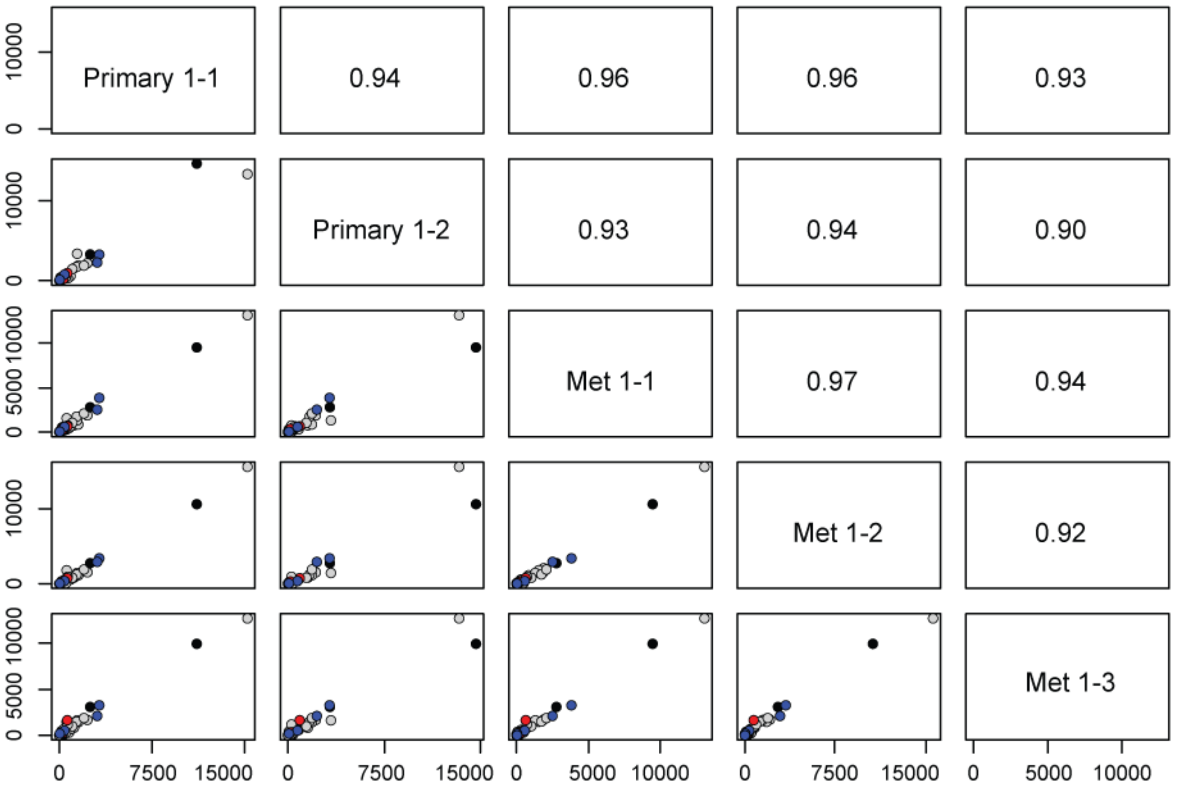

Patient 2 ($RNA^{mut\ allele}$ read count [normalized]):

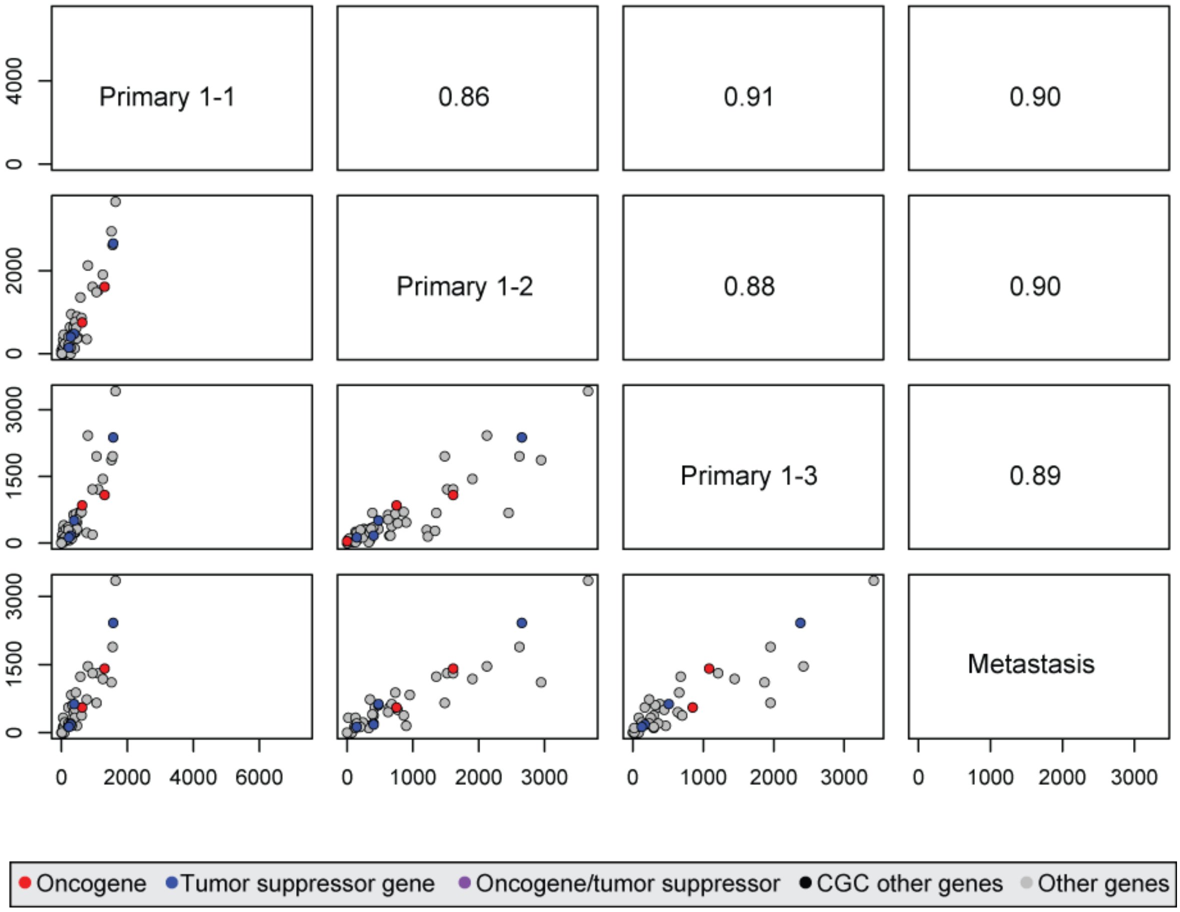

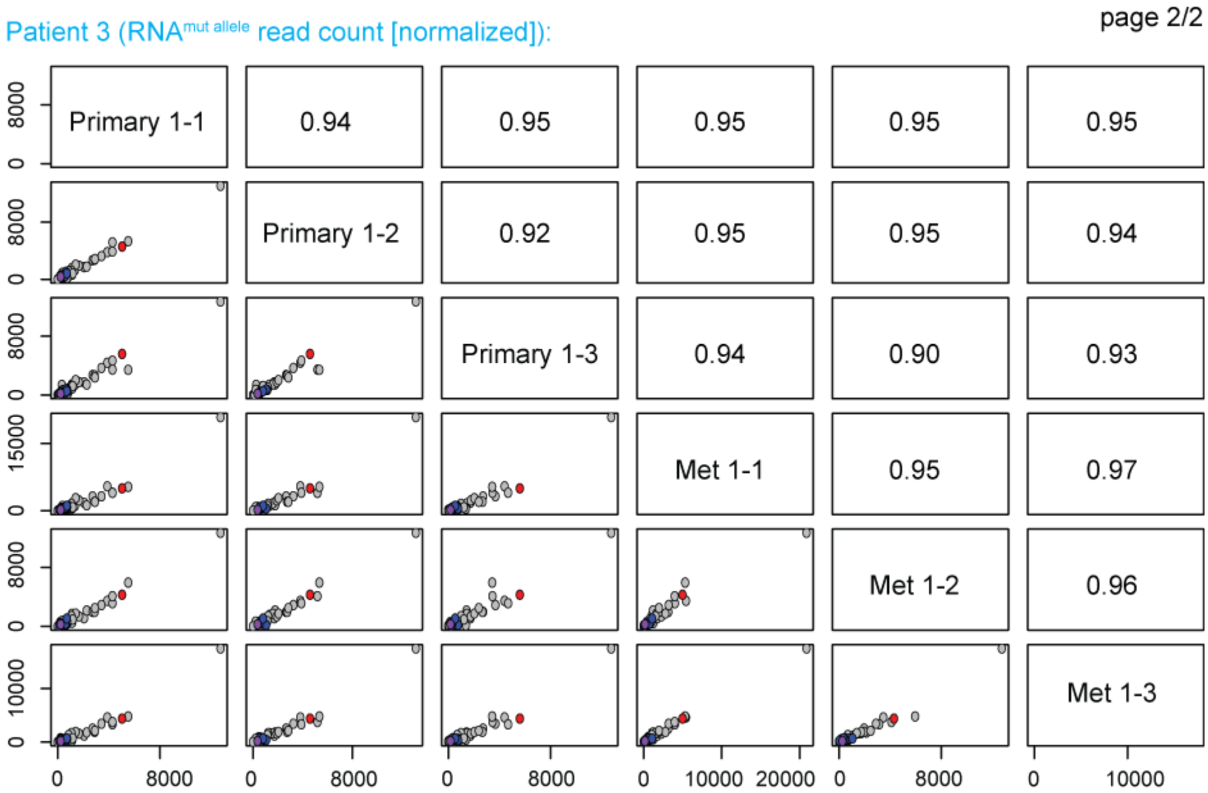

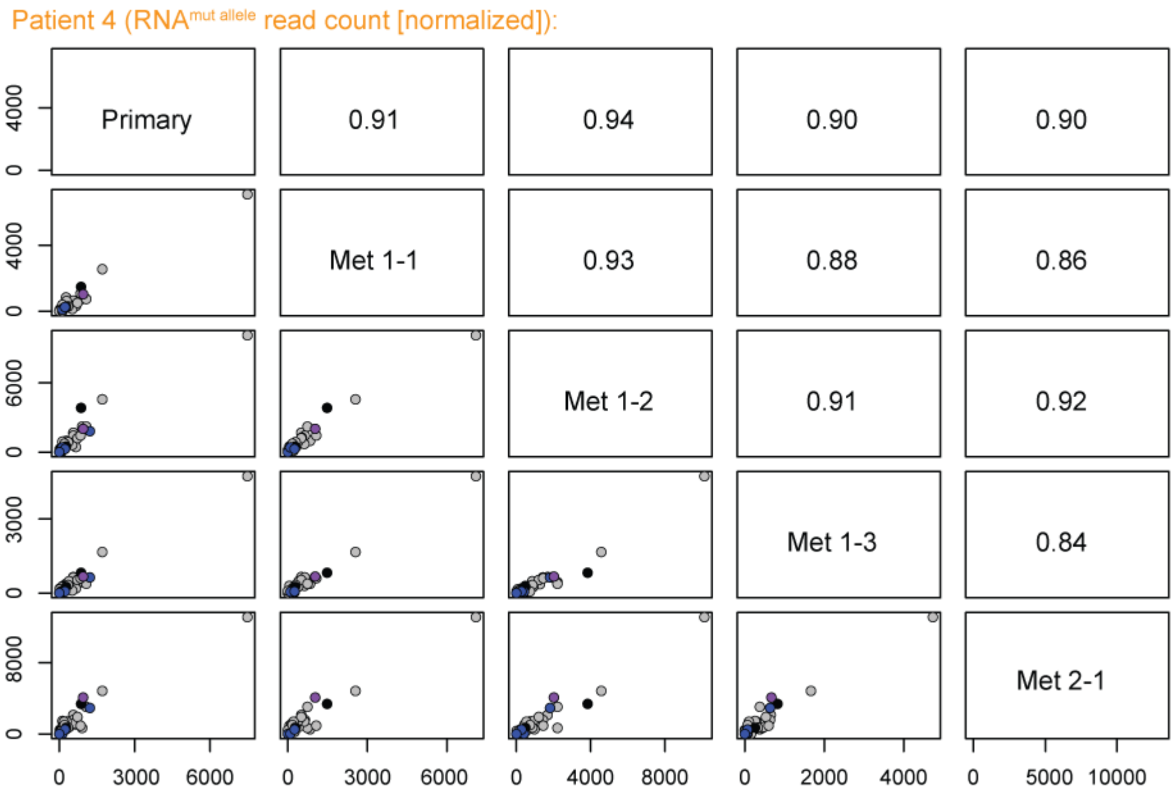

Figure S23. Comparisons of RNA read counts of mutated alleles among multiple tumor samples from each of 4 patients

Each scatter plot matrix represents pair-wise comparisons of tumor samples from each of 4 patients, with normalized RNA read counts of the mutated alleles plotted on both the vertical and horizontal axes (bottom left panels), and with the corresponding Spearman's ρ indicated in the upper right

panels. Each dot represents a mutation, colored according to target gene category (only mutations found in expressed genes are plotted). The proportionality of mutation expression levels between sample pairs was high in all patients, with median Spearman's ρ of 0.94 (95% CI 0.92-0.96; patient 1), 0.90 (95% CI 0.86-0.91; patient 2), 0.95 (95% CI 0.94-0.95; patient 3), and 0.91 (95% CI 0.86-0.93; patient 4).
